# Hydrogels as Local Structural-Protective Platforms in Rheumatoid Arthritis: An Evidence-Graded Review Across the Synovium–Cartilage–Bone Axis

**DOI:** 10.3390/gels12070601

**Published:** 2026-07-06

**Authors:** Ruiqi Liao, Kailang Mu, Fei Ran, Lixia Yang, Yunqian Feng, Tianrui Xu, Xuemei Zhong, Fudao Wei, Yuxin Pang, Gang Liu, Yuchen Liu

**Affiliations:** 1 College of Pharmacy, Guizhou University of Traditional Chinese Medicine, Guiyang 550025, China; m18285888952_1@163.com (R.L.); mkl980818@163.com (K.M.); rf991125@163.com (F.R.); 18985546954@163.com (L.Y.); yunqian0911@163.com (Y.F.); xtr0506@163.com (T.X.); 15086009935@163.com (X.Z.); w3161245891@163.com (F.W.); pyxmarx@126.com (Y.P.); 2 College of Pharmacy, Guangdong Medical University, Dongguan 523808, China

**Keywords:** rheumatoid arthritis, hydrogel, synovium–cartilage–bone axis, intra-articular delivery, local immunomodulation, cartilage protection, osteoimmune regulation, structural disease modification

## Abstract

Rheumatoid arthritis (RA) is a systemic autoimmune disease in which persistent synovitis drives interconnected cartilage degradation, bone erosion, and functional decline. Conventional synthetic, biologic, and targeted synthetic disease-modifying antirheumatic drugs (DMARDs) remain the foundation of RA management. Hydrogel-based local therapy should therefore be positioned as an adjunct for selected joints rather than as a substitute for systemic disease control. Hydrogels provide a versatile local materials platform because their injectability, tunable crosslinking, tissue retention, stimulus-responsive release, interfacial adhesion, lubrication, and extracellular matrix-mimetic properties can be tailored to the inflamed joint microenvironment. This narrative, evidence-graded review evaluates local hydrogel therapies using two complementary frameworks: the synovium–cartilage–bone pathological axis and a materials-science chain linking composition and crosslinking to structure and properties, release and degradation, and tissue-level outcomes. Evidence is classified as direct RA evidence, transferable evidence from related disease or tissue-engineering models, or conceptual evidence from mechanistic and materials-science studies. Therapeutic outcomes are separately graded as local immunomodulation, structural protection, tissue repair, or functionally validated structural disease modification. Current preclinical evidence supports the use of hydrogels for sustained local delivery and synovial immunomodulation, while selected systems demonstrate cartilage-protective or anti-erosive effects. However, durable multitissue restoration accompanied by functional recovery remains insufficiently demonstrated. Future studies should prioritize RA-relevant long-term models, in vivo intra-articular pharmacokinetics and biodistribution, standardized structural and functional endpoints, repeat-dose safety, and evaluation as add-on therapy to systemic DMARDs.

## 1. Introduction

Rheumatoid arthritis (RA) is a systemic autoimmune disease characterized by chronic synovitis that can lead to cartilage loss, bone erosion, joint deformity, and functional impairment [1]. Treat-to-target strategies based on conventional synthetic, biologic, and targeted synthetic disease-modifying antirheumatic drugs (DMARDs) have substantially improved disease control and patient outcomes [2,3]. Nevertheless, heterogeneity in therapeutic response, dose-limiting adverse effects, and discordance between systemic disease activity and pathology in individual joints mean that some patients continue to experience residual synovitis or progressive local structural damage [4,5]. Intra-articular administration can increase local drug exposure and may reduce systemic exposure; however, unformulated agents are frequently limited by dilution in synovial fluid, lymphatic clearance, and insufficient tissue retention, resulting primarily in short-lived control of inflammation and symptoms [6,7]. Local biomaterial platforms should therefore be developed as adjuncts within a treat-to-target strategy, with the aims of prolonging local drug exposure, modulating the joint microenvironment, and protecting vulnerable tissues in selected joints. Plausible near-term indications include persistent inflammation in one or a few joints despite otherwise adequate systemic therapy and the need for prolonged intra-articular drug exposure, focal cartilage-surface protection, and local anti-erosive treatment. Comparative and add-on studies with systemic DMARDs are required before claims of systemic dose sparing, prevention of radiographic progression, or disease modification can be justified.

Previous reviews of hydrogel therapies for RA and other arthritides have mainly organized the field according to polymer origin, crosslinking chemistry, route of administration, or stimulus-responsive release [8,9]. These frameworks are valuable for describing material fabrication and drug-delivery mechanisms, but they do not consistently distinguish among four clinically different outcomes: local immunomodulation, structural protection, tissue repair, and functionally validated structural disease modification. The present review differs in two principal respects. First, it organizes the evidence around pathological coupling across the synovium–cartilage–bone axis rather than treating synovitis, cartilage injury, and bone erosion as independent outcomes. Second, it separates the directness of disease evidence from the level of therapeutic outcome, thereby preventing direct RA data from being conflated with findings from osteoarthritis, bone-defect, wound-healing, or general tissue-engineering models. This framework enables readers to identify both which hydrogel strategies have been evaluated directly in RA-relevant settings and what level of therapeutic claim is supported by the reported endpoints.

Joint damage in RA arises from mutually reinforcing pathological interactions among the synovium, cartilage, and bone rather than from independent lesions in three separate tissue compartments. Synovial macrophages and FLS continuously produce cytokines, chemokines, and matrix-degrading enzymes that promote cartilage matrix loss. In turn, cartilage degradation products, impaired lubrication, and abnormal mechanical stimulation further amplify synovial inflammation. Activation of osteoclasts mediated by receptor activator of nuclear factor-κB ligand (RANKL) causes bone erosion and compromises the mechanical stability of the osteochondral unit [10,11]. Given this pathological coupling, reductions in inflammatory mediators, paw swelling, or short-term arthritis scores alone are insufficient to demonstrate structural disease modification. Similarly, preserved cartilage staining does not necessarily indicate regeneration, and reductions in osteoclast-related markers do not equate to the repair of bone erosions. Higher-level evidence requires the combined demonstration of structural preservation by histological or imaging analyses, new tissue formation, integration with host tissues, and sustained functional benefits [12,13].

Against this background, this review critically synthesizes research on local hydrogel-based interventions for RA by using the synovium–cartilage–bone axis as the disease-biological framework and the relationship among hydrogel “composition and crosslinking—structure and properties—release and degradation—tissue effects” as the materials-science framework. Three aspects are evaluated. First, we examine how different hydrogel designs affect intra-articular retention, responsiveness to pathological cues, and interactions with tissue interfaces. Second, we assess the level of evidence achieved for synovial immunomodulation, cartilage protection or repair, and control of bone erosion. Third, we identify the experimental and translational evidence that remains necessary to establish durable, functionally meaningful structural disease modification across multiple tissues [14,15,16]. A synthesis of the available literature suggests that the most established value of hydrogels remains sustained local delivery and modulation of the immune microenvironment. By contrast, systems that simultaneously target the synovium, cartilage, and bone while restoring joint function remain at an early preclinical stage.

## 2. Literature-Search Strategy and Evidence Classification

This review was designed as a narrative, evidence-graded synthesis rather than as a formal systematic review or meta-analysis. The principal databases searched were PubMed/MEDLINE, Web of Science Core Collection, Scopus, and Embase. Google Scholar, CNKI, and WanFang were used as supplementary sources. Searches covered studies published between 1 January 2010 and 9 June 2026. Seminal studies published before 2010 were identified through backward citation tracking and were included only when they established essential principles of RA pathology or hydrogel design. Representative search combinations integrated disease-related terms (“rheumatoid arthritis”, “inflammatory arthritis”, “synovitis”, “cartilage destruction”, “bone erosion”, “osteoclast”, and “fibroblast-like synoviocyte”), material-related terms (“hydrogel”, “injectable hydrogel”, “in situ gel”, “thermosensitive”, “hyaluronic acid”, “gellan gum”, “chitosan”, “GelMA”, “supramolecular”, “responsive”, and “nanocomposite”), and outcome-related terms (“intra-articular retention”, “pharmacokinetics”, “cartilage protection”, “bone repair”, “osteogenesis”, “lubrication”, and “functional recovery”). Reference lists of relevant reviews and eligible primary studies were also screened.

Peer-reviewed studies were included when they described a hydrogel composition or crosslinking strategy, involved local delivery or local tissue interaction, and reported at least one interpretable biological, pharmacokinetic, structural, or functional endpoint relevant to the review question. Studies were excluded from the efficacy synthesis when they lacked a hydrogel platform, involved only systemic administration without a local-material rationale, or provided insufficient methodological information to interpret the material or biological findings. Studies reporting only generic in vitro anti-inflammatory activity were not used as evidence of RA efficacy, although they could be cited as conceptual evidence for a mechanism or material-design principle. Studies from osteoarthritis, bone-defect, wound-healing, inflammatory, or general tissue-engineering models were retained only when they provided transferable design evidence and were explicitly identified as non-RA evidence. Review articles were used to contextualize the field but were not treated as primary evidence of hydrogel efficacy.

Evidence directness and therapeutic outcome were graded independently. Direct RA evidence was defined as evidence generated using human RA-derived cells or tissues, clinical RA samples, or established RA-relevant experimental inflammatory arthritis models, such as collagen-induced arthritis, antigen-induced arthritis, or K/BxN serum-transfer arthritis. Transferable evidence was derived from osteoarthritis, bone-defect, wound-healing, general inflammation, or tissue-engineering models and was used only to support material-design or mechanistic rationale. Conceptual evidence comprised materials-science or mechanistic studies without disease-specific validation. Classification was applied at the level of the individual claim rather than automatically at the level of the entire publication. A single study could therefore contribute to different categories for different claims, but each statement was assigned the most conservative category directly supported by the relevant model and endpoint (Table 1).

## 3. Pathological Coupling of the Synovium–Cartilage–Bone Axis in RA

**Figure 1:**
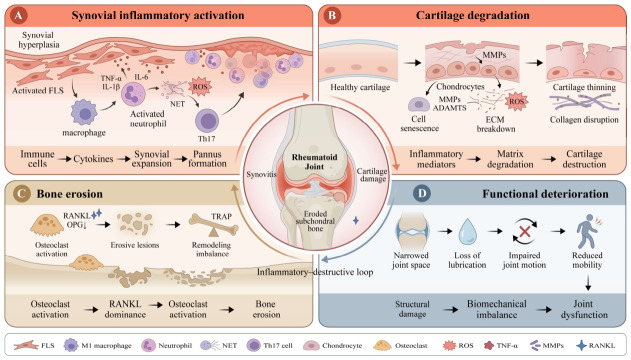
Pathological coupling across the synovium–cartilage–bone axis in rheumatoid arthritis. (**A**) Activated macrophages, FLS, neutrophils, NETs, Th17 cells, reactive oxygen species, and inflammatory cytokines promote synovial hyperplasia and pannus formation. (**B**) Inflammatory mediators and matrix-degrading enzymes impair chondrocyte function, fragment the extracellular matrix, and accelerate cartilage loss. (**C**) RANKL/OPG imbalance promotes osteoclast differentiation and progressive bone erosion. (**D**) The resulting structural damage impairs lubrication, joint motion, load distribution, and mobility. Reciprocal interactions among these compartments sustain an inflammatory–destructive loop and provide the biological rationale for multitissue evaluation of local hydrogel interventions.

An RA joint does not consist of independent lesions involving synovitis, cartilage damage, and bone erosion. Instead, it represents a coupled system sustained by immune signaling, matrix degradation, abnormal mechanical forces, and bone remodeling. This section focuses on pathological nodes that can be directly translated into hydrogel design requirements: the synovial macrophage–FLS network, disruption of the cartilage matrix and lubrication barrier, and RANKL-mediated osteoclast activation. Defining the causal relationships among these tissue compartments is essential for determining whether a hydrogel merely prolongs local drug exposure or has the potential to protect multiple joint tissues from structural damage. The pathological coupling among synovial inflammation, cartilage degradation, bone erosion, and functional deterioration is summarized in Figure 1.

### 3.1. The Engine of Synovial Inflammation: The Macrophage–FLS Network

Macrophages and fibroblast-like synoviocytes (FLSs) form a major inflammatory and tissue-destructive network in the RA synovium. Although conventional M1/M2 markers are frequently used to describe macrophage responses, synovial macrophages occupy heterogeneous and dynamically regulated states. Pro-inflammatory macrophage populations release TNF-α, IL-1β, IL-6, and other mediators that activate NF-κB- and MAPK-associated signaling in FLS, thereby promoting FLS proliferation, migration, and inflammatory activity [17,18,19].

Activated FLSs are the principal effector cells linking synovial inflammation to structural destruction. In addition to inflammatory stimulation, intracellular regulatory mechanisms, such as methyltransferase-like 3 (METTL3)-mediated N6-methyladenosine (m6A) modification, can maintain their invasive phenotype [20]. RA-derived FLSs (RA-FLS) secrete matrix metalloproteinase-1 (MMP-1), matrix metalloproteinase-3 (MMP-3), matrix metalloproteinase-9 (MMP-9), matrix metalloproteinase-13 (MMP-13), and a disintegrin and metalloproteinase with thrombospondin motifs 5 (ADAMTS-5), which directly degrade type II collagen and aggrecan. RA-FLSs also express RANKL and thereby promote osteoclast differentiation [21,22]. Signaling associated with basigin (CD147) can additionally upregulate vascular endothelial growth factor (VEGF) and matrix-degrading enzymes, promoting synovial angiogenesis and facilitating the continuous recruitment of inflammatory cells [23]. Therefore, reductions in soluble cytokine levels alone are insufficient to demonstrate the restoration of synovial homeostasis. FLS invasion, pannus formation, and intercellular crosstalk should be evaluated as endpoints more directly linked to structural damage.

At the synovium–cartilage interface, hyperplastic synovial tissue forms a pannus enriched in macrophages and invasive FLS, which extends along the cartilage surface and osteochondral interface [24]. The coexpression of CD147 with MMP-1 and matrix metalloproteinase-2 (MMP-2) at the cartilage–pannus junction, together with the promotion of FLS migration by C-C motif chemokine ligand 18 (CCL18), further demonstrates the tissue-invasive nature of synovial pathology [25,26]. For hydrogel systems, this pathological feature indicates that the design objective should not be limited to creating an intra-articular drug depot. Synovial adhesion, targeting of inflammatory cells or FLS, and sustained modulation of the macrophage–FLS feedback loop should also be considered.

### 3.2. Cartilage Matrix Degradation and Impaired Lubrication

Cartilage damage is jointly driven by biochemical catabolism and deterioration of interfacial mechanics. IL-1β and TNF-α suppress the synthesis of type II collagen and aggrecan by chondrocytes while inducing the expression of matrix-degrading enzymes, including MMP-13 and ADAMTS-5 [27,28]. Inflammation-associated metabolic reprogramming further compromises the anabolic capacity and stress tolerance of chondrocytes [29]. Regulatory axes involving secreted protein acidic and rich in cysteine-like 1 (SPARCL1) and plexin C1 (PLXNC1) have been identified primarily in studies of osteoarthritis or inflammation-stimulated chondrocytes. They may therefore serve as transferable mechanistic evidence linking inflammation to matrix degradation but cannot be regarded as direct evidence of hydrogel efficacy in RA [30,31].

Synovitis also alters the abundance, molecular state, and interfacial functions of hyaluronic acid (HA) and lubricin, also known as proteoglycan 4 (PRG4), in synovial fluid. An imbalance between HA synthesis and degradation, reduced PRG4 levels, and disruption of the phospholipid layer impair hydration lubrication, thereby increasing friction and shear stress at the articular surface [32,33,34,35]. Mechanical stimulation subsequently promotes the release of inflammatory mediators by chondrocytes and synovial cells, establishing an “inflammation–friction–matrix damage” feedback loop. Meanwhile, the accumulation of reactive oxygen species (ROS) and mitochondrial dysfunction can induce chondrocyte apoptosis or senescence, further limiting matrix renewal [36]. Evaluations of cartilage-directed hydrogels should therefore encompass anticatabolic activity, cell survival, matrix preservation, and tribological performance. A reduction in inflammatory mediators alone cannot be equated with cartilage regeneration.

### 3.3. Bone Erosion Driven by RANKL/OPG Imbalance

Osteoclasts are the direct executors of bone erosion in RA, and RANKL–receptor activator of nuclear factor-κB (RANK) signaling is the central pathway governing their formation and activation. Both synovial FLS and activated T cells can express RANKL. However, cell-specific knockout studies indicate that FLS-derived RANKL is particularly important for local osteoclast formation and bone erosion in inflammatory arthritis [37]. After RANKL binds to RANK on the surface of osteoclast precursors, signaling through tumor necrosis factor receptor-associated factor (TRAF) proteins, cellular Src tyrosine kinase (c-Src), c-Jun N-terminal kinase (JNK), and NF-κB induces nuclear factor of activated T cells 1 (NFATc1) and Fos proto-oncogene (c-Fos), thereby initiating the osteoclast differentiation program [38,39].

Osteoprotegerin (OPG) acts as a soluble decoy receptor that limits the binding of RANKL to RANK. In RA synovial fluid and diseased tissues, the RANKL/OPG balance shifts toward osteoclastogenesis and is closely associated with local bone loss [40,41,42]. Studies of combined fluoride–arsenic exposure and simvastatin further indicate that this ratio can be modulated by exogenous factors. However, these findings do not constitute direct evidence from RA hydrogel studies and can only support the general modifiability of this pathway [43,44]. Bone protection should therefore be evaluated preferentially using tartrate-resistant acid phosphatase (TRAP)-positive cells, cathepsin K (CTSK) expression, bone erosion area, and micro-computed tomography (micro-CT) parameters in RA models. Changes in RANKL/OPG expression alone should not be used as substitutes for structural endpoints.

Mature osteoclasts accumulate at the cartilage–bone junction and erosion surfaces, where they degrade the bone matrix through TRAP, CTSK, MMP-9, and related factors [45,46]. NFATc1 and c-Fos regulate their maturation and bone-resorptive activity [47,48]. Osteocyte-derived RANKL and auxiliary pathways, including Notch signaling, may also contribute to local bone remodeling, although their relative contributions at different stages of RA remain to be defined [49,50]. Clinically, RANKL blockade can inhibit the progression of bone erosion but cannot replace systemic control of synovial inflammation [51]. These findings indicate that bone-directed hydrogels should distinguish among three levels of evidence: “inhibition of osteoclast-mediated bone resorption,” “preservation of bone structure,” and “new bone formation.” Their complementary relationship with anti-inflammatory therapy should also be evaluated.

### 3.4. Multitissue Positive Feedback and Its Implications for Hydrogel Design

The synovium, cartilage, and bone are interconnected through soluble mediators, matrix fragments, and abnormal mechanical forces. TNF-α, IL-1β, and matrix metalloproteinases (MMPs) released by synovial cells promote cartilage matrix loss. In turn, degradation products, including fibronectin fragments and collagen peptides, amplify synovial inflammation through pathways involving Toll-like receptors (TLRs) and NOD-like receptor family pyrin domain-containing 3 (NLRP3) [8,52]. Meanwhile, synovium-derived RANKL promotes bone erosion, weakens subchondral bone support, and alters the distribution of joint loads [9]. Inflammation, matrix degradation, and bone resorption therefore do not necessarily occur in a strictly sequential manner; rather, they may proceed concurrently and reinforce one another within the same joint.

Calcium salts, osteopontin, and other matrix-derived signals released from areas of bone erosion may further influence the activity of synovial cells, chondrocytes, and osteoclasts. Shared pathways, such as interleukin-6/Janus kinase 2/signal transducer and activator of transcription 3 (IL-6/JAK2/STAT3) signaling, provide a molecular basis for signal amplification across tissue compartments [53]. However, several proposed bone-derived feedback mechanisms are still based primarily on in vitro studies or non-RA models. At present, they should therefore be considered pathological hypotheses requiring validation rather than evidence that multitissue hydrogel systems have already been proven effective.

This coupled network imposes three direct requirements on material design. First, cargo release should be matched to the principal pathological compartment and its accessibility. The synovium is relatively accessible, whereas the cartilage and bone interfaces present stronger diffusion and binding barriers [54,55]. Second, multitarget therapy should not be equated with simply increasing the number of therapeutic cargos. Spatial targeting, disease-triggered release, or sequential release should be used to minimize mutual interference. Third, blockade of a single pathway may leave alternative inflammatory or osteoclastogenic signals intact. Therapeutic evaluation must therefore include endpoints in the synovium, cartilage, and bone [56]. Accordingly, the following sections on hydrogels focus on how material composition, crosslinking, interfacial interactions, degradation, and release behavior determine whether a system can disrupt these feedback loops rather than merely prolong local drug exposure.

## 4. Hydrogel Design Requirements for Local Structural Protection and Disease-Modifying Potential

**Figure 2:**
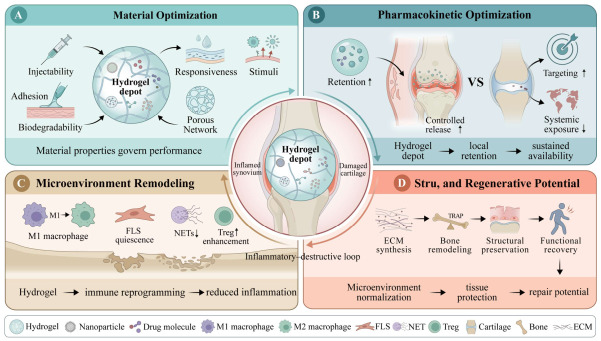
Hydrogel-based intervention strategies for rheumatoid arthritis joints. Hydrogels can serve as local intra-articular depots that integrate material engineering, pharmacokinetic optimization, immune microenvironment remodeling, and structural protection. (**A**) Injectable, adhesive, biodegradable, porous, and stimulus-responsive hydrogel networks determine local retention, tissue interaction, degradation, and release behavior. (**B**) Hydrogel depots can prolong intra-articular retention, enable controlled release, enhance local exposure, and reduce systemic exposure compared with unformulated drugs. (**C**) Local hydrogel systems may remodel the inflammatory microenvironment by modulating macrophage states, suppressing fibroblast-like synoviocyte activation, reducing NET burden, and promoting regulatory immune responses. (**D**) These effects may contribute to extracellular matrix preservation, bone remodeling, structural protection, and functional recovery. The regenerative potential of such systems should be interpreted cautiously and distinguished from definitive evidence of tissue regeneration.

The ability of hydrogels to promote structural disease modification in RA depends not on the simple accumulation of functional modules, but on whether their material properties are matched to the intended application. Intra-articular drug depots, cartilage-surface lubricating layers, scaffolds for cartilage defects, and filling materials for bone erosions have distinct design requirements. This section discusses material parameters that can be translated into biological effects from four perspectives: delivery and retention, interfacial properties and dynamic stability, responsiveness to the pathological microenvironment, and the coupling of mechanics, degradation, and release. The major hydrogel design strategies that connect material properties with pharmacokinetic behavior, immune microenvironment remodeling, and structural protection are summarized in Figure 2.

### 4.1. Injectability, In Situ Gelation, and Intra-Articular Retention

Injectability is jointly determined by precursor viscosity, shear-thinning behavior, needle gauge, injection rate, joint size, and cargo properties. Therefore, neither a single viscosity value nor a specific needle gauge should be regarded as a universal standard. In situ gelation requires a balance between a practical working time and rapid immobilization after injection. Gelation that occurs too quickly may cause gel formation within the needle, whereas excessively slow gelation increases the risks of dilution by synovial fluid and leakage from the injection site. Previous reviews have systematically summarized the rheological and gelation requirements of injectable hydrogels for the delivery of drugs and biological macromolecules [57,58,59,60]. Thermosensitive polymers and poloxamers can undergo a body-temperature-triggered sol–gel transition [61,62]. Networks mediated by ions or particles may improve local retention [63]. Horseradish peroxidase (HRP)-catalyzed coupling of phenolic groups enables the gelation rate and network strength to be adjusted under mild conditions, although oxidant concentration and residual enzymatic activity must be considered in cytocompatibility assessments [64,65].

Intra-articular retention should be measured directly through imaging-based tracking, tissue-distribution analyses, and local pharmacokinetic studies rather than evaluated against a predefined “ideal” retention period. Free proteins and nanoparticles may be rapidly cleared through synovial fluid exchange and lymphatic drainage [66]. However, excessively prolonged material residence may increase foreign-body reactions or interfere with tissue remodeling. Multiblock copolymer microspheres have demonstrated long-term retention in animal joints [67], whereas hydrogels loaded with sinomenine-containing liposomes or nanoformulated iguratimod provide examples of sustained local delivery in RA models [68,69]. Injection force, gelation time, gel stability after dilution with synovial fluid, in vivo retention, degradation, and cargo exposure should therefore be reported together. Intra-articular efficacy should not be inferred solely from in vitro release profiles.

A central translational gap is that in vitro cumulative-release curves cannot predict intra-articular residence time, synovial-fluid dilution, lymphatic clearance, penetration into synovium or cartilage, or drug concentration at erosion surfaces. Accordingly, future studies should include in vivo concentration–time profiles of both cargo and carrier, imaging or quantitative tracking of material degradation, biodistribution in synovium/cartilage/bone/draining lymph nodes/plasma, and safety after repeated administration. For responsive systems, the pathological trigger should be verified within the joint rather than inferred from buffer-based release experiments.

### 4.2. Adhesion, Self-Healing, Fatigue Stability, and Lubrication

Interfacial properties should be determined by the intended function of the hydrogel. A simple drug depot primarily needs to resist washout by synovial fluid without restricting joint movement. Materials designed for the cartilage surface must additionally provide wet-tissue adhesion, low friction, and stability under cyclic loading. Scaffolds intended for osteochondral defects place greater emphasis on shape conformity, cell adhesion, and tissue integration. Integrin-binding sequences, such as arginine–glycine–aspartic acid (RGD), and the temporally controlled presentation of osteogenic peptides can promote cell attachment or differentiation. However, the relevant evidence is derived mainly from osteoarthritis and general tissue-engineering studies and therefore represents transferable design evidence [70,71]. By contrast, MMP-binding hydrogels have reduced synovial inflammation and improved cartilage-related outcomes in RA models, providing direct evidence for a material-design rationale based on “interfacial localization–pathological factor capture–structural protection” [72].

Dynamic covalent bonds and reversible noncovalent interactions can impart shear-thinning and self-healing properties to hydrogels. However, self-healing behavior cannot replace fatigue testing under physiological loading. Injectable hydrogels designed to degrade neutrophil extracellular traps [73] and ROS/pH-responsive composite hydrogels containing polymeric micelles [74] demonstrate that dynamic networks can combine injectability, local immobilization, and sustained delivery. The swelling resistance and release behavior of enzymatically crosslinked tyramine–gellan gum hydrogels [75], the photothermal and mechanical functions of black phosphorus nanosheet-containing systems [76], and the regulation of macrophage-derived extracellular vesicles by in situ pore-forming hydrogels [77] further show that network architecture can simultaneously affect material stability and cellular responses. Systems intended to function at the cartilage interface should also be evaluated for wet-state adhesion, coefficient of friction, and performance retention after cyclic compression or shear. These parameters, however, should not be extrapolated as universal requirements for all intra-articular hydrogels.

### 4.3. Responsiveness to Pathological Microenvironmental Signals, Including ROS, pH, and MMPs

RA joints may exhibit increased oxidative stress, local acidification, and elevated MMP activity. However, these signals vary among patients, disease stages, and tissue compartments. The design of responsive hydrogels should therefore prioritize validation of whether the triggering threshold covers the actual pathological range, whether the material remains stable under normal joint conditions, and whether release becomes reversible or terminates spontaneously after disease remission. ROS-sensitive structures, including thioethers, thioketals, and boronate esters, can be incorporated to mediate network degradation or cargo release. Studies of brain injury and cutaneous wounds have demonstrated the feasibility of these chemical mechanisms, but they provide only cross-disease materials-science evidence [78,79]. In RA models, dual dynamically crosslinked hydrogels have enabled ROS modulation together with sustained release of triptolide [80]. Nanozymes such as cerium oxide can provide both ROS-scavenging and catalytic activities [81,82]. However, the non-RA wound and skin-inflammation studies cited in [55,81,82] primarily support the design principles of nanozyme–hydrogel systems and do not directly demonstrate efficacy in RA.

pH-responsive systems should avoid treating a single acidic range as a fixed feature of all RA joints. A more appropriate approach is to compare swelling, degradation, and release under physiological pH and several mildly acidic conditions. MMP-responsive networks can convert pathological protease activity into local cargo release or factor capture through cleavable peptide sequences or protein-binding sites. Studies using ROS- and MMP-responsive microneedles for pathological scars demonstrate the engineering feasibility of multiresponsive systems. However, their therapeutic objective of promoting collagen degradation differs from that of protecting cartilage in RA [83]. Multiresponsive designs should therefore be regarded only as transferable concepts until they have been revalidated in RA models. Existing reviews have summarized the application boundaries of injectable responsive hydrogels in osteoarthritis (OA) and RA [84], whereas metabolically driven bioresponsive hydrogels provide direct examples of exploiting hypoxia and enzymatic environments for on-demand treatment in RA [85].

### 4.4. Coupled Design of Mechanical Properties, Degradation, and Release Behavior

Mechanical properties, degradation, and release are not independent parameters. Increasing crosslinking density generally enhances modulus and retention but may reduce injectability, slow tissue ingrowth, and restrict the diffusion of macromolecules. Material design should therefore begin by distinguishing among application scenarios. Intra-articular drug depots require moderate compliance, stable retention, and minimal interference with joint movement. Cartilage-surface lubricating layers require wet adhesion and low friction. Osteochondral scaffolds require greater load-bearing capacity and tissue integration. Storage modulus, compressive modulus, and tissue Young’s modulus should not be treated as equivalent, and a conventional drug depot does not need to mechanically match bone tissue. Accordingly, the degradation period should be determined by the therapeutic window of the cargo, the planned repeat-dosing schedule, and whether the hydrogel serves a scaffold function rather than being uniformly set to a predefined number of weeks.

The release pattern should likewise be determined by the mechanism of the cargo. Anti-inflammatory small molecules may need to reach an effective concentration rapidly, whereas proteins, nucleic acids, and regenerative factors depend more strongly on the preservation of biological activity and sustained exposure. An “initial burst followed by sustained release” is therefore not the default optimal profile for every system. Dual-drug intra-articular delivery systems have demonstrated the importance of cargo ratios and synchronized release for synergistic efficacy [86]. The encapsulation of prednisolone liposomes in HA hydrogels shows that a secondary carrier can reduce diffusion and prolong local exposure [87]. At a minimum, studies should report network composition and crosslinking density, wet-state rheological and mechanical properties, degradation assessed by changes in mass and volume, cargo activity, complete release profiles, and in vivo pharmacokinetics. These material parameters should also be linked to synovial, cartilage, bone, and functional endpoints.

The implementation of these material parameters varies substantially among studies providing direct evidence in RA. To facilitate comparisons of hydrogel matrices, gelation methods, functional cargos, delivery mechanisms, and application scenarios, Table 2 summarizes representative local hydrogel platforms supported by direct experimental evidence in RA. Overall, existing systems have progressed from simple sustained drug release to pathological responsiveness, interfacial adhesion, lubrication, and coordinated multicomponent delivery. However, increasing the number of material functions does not necessarily produce structural benefits across multiple tissues. The following sections therefore evaluate biological effects separately in the synovial, cartilage, and bone compartments.

## 5. Local Modulation of the Synovial Inflammatory Microenvironment

### 5.1. Modulation of Macrophage States and Functions

Macrophages in the RA synovium contribute to cytokine amplification, antigen presentation, matrix degradation, and osteoclastogenesis and are therefore among the most frequently targeted immune cells in local hydrogel-based interventions. The M1/M2 framework provides a convenient summary of proinflammatory and repair-associated states, but it does not fully capture the continuous, reversible, and tissue compartment-dependent spectrum of macrophage phenotypes in the RA synovium. Hydrogel studies should therefore not regard reduced CD86 expression or increased CD206 expression alone as sufficient evidence of “immune reprogramming.” Inflammatory mediators, phagocytic and metabolic states, synovial histopathology, and cartilage and bone outcomes should also be evaluated [102,103].

Mitochondrial function and energy metabolism can either constrain or facilitate transitions between macrophage states, providing a mechanistic basis for using local materials to regulate glycolysis, oxidative phosphorylation, and redox balance [104,105]. However, a substantial proportion of the available supporting evidence is derived from models of uterine scarring, fatty liver disease, graft survival, or other inflammatory conditions. Examples include thermosensitive hydrogels loaded with cells and asiaticoside, macrophage-targeted fullerenes, and metabolic regulatory strategies [106,107,108,109,110,111,112]. Although the associated material designs may be transferable, they do not directly demonstrate efficacy in RA. RA-related studies further suggest that lactate, protein lactylation, and macrophage metabolic reprogramming may serve as targets for local intervention [113,114]. Future systems should clearly distinguish the intrinsic immunomodulatory effects of the material from those of the encapsulated therapeutic agents. Their disease-modifying value should then be validated by sustained structural benefits across multiple joint tissues.

### 5.2. Inhibition and Selective Targeting of Pathological FLS

FLSs exhibit persistent activation, migration, and matrix-invasive behavior and are key effector cells linking synovitis to cartilage and bone destruction. MicroRNA-221/222 (miR-221/222), apoptosis signal-regulating kinase 1 (ASK1), and pathways including NF-κB and MAPK regulate FLS proliferation, inflammatory mediator production, and apoptosis, providing direct mechanistic evidence from RA for the local delivery of nucleic acids or small-molecule inhibitors [115,116,117]. However, most of these studies did not investigate hydrogel systems. A more accurate interpretation is therefore that hydrogels may convert established FLS-targeting molecules into local therapeutic strategies by increasing intra-articular retention and limiting systemic exposure, rather than that hydrogels have already been shown to selectively eliminate pathological FLS.

Fibroblast activation protein inhibitor (FAPI)-modified nanoparticles and theranostic nanomedicines activated by hypoxic or acidic environments have shown the potential to suppress or ablate FLS in arthritis models [118,119]. However, their carrier properties and safety boundaries cannot be directly extrapolated to hydrogels. Complete elimination of FLS may also impair the synovial barrier, nutrient exchange, and synovial fluid homeostasis. Suppression of the invasive phenotype is therefore generally more consistent with translational requirements than nonselective cell ablation. The lineage overlap between synovial mesenchymal stem cells (MSCs) and FLS, together with the potentially bidirectional effects of platelet-rich plasma on FLS—either suppressing inflammation or promoting matrix degradation—also indicates that evidence supporting post-ablation regenerative strategies remains insufficient [120,121,122]. Within the evidentiary framework of this review, these approaches should be classified as conceptual or early translational strategies. Evaluation should include cell-subpopulation specificity, long-term synovial function, and the risk of disease recurrence.

### 5.3. Coordinated Regulation of Macrophage–FLS Crosstalk

Macrophages and FLS form a bidirectional amplification network through TNF-α, IL-1β, chemokines, metabolites, and extracellular vesicles (EVs). RA coculture models have shown that direct contact and paracrine signaling between these cell types jointly enhance FLS invasion and the proinflammatory state of macrophages [123]. The ability of FLS-derived exosomes to promote macrophage glycolysis was initially demonstrated in OA models and should therefore be regarded as a transferable mechanism [124]. By contrast, RA studies support roles for FLS-derived exosomes and hyaluronan and proteoglycan link protein 1 (HAPLN1) in promoting macrophage migration or proinflammatory polarization [114,125]. These findings indicate that reporting macrophage polarization or FLS proliferation alone is insufficient to determine whether the pathogenic synovial network has been genuinely reset [126].

The principal value of hydrogels in this context is their ability to confine cargos acting on different cell types within the same local compartment and to reduce drug–drug interference through controlled-release sequences. For example, exosomes or microRNAs (miRNAs) may be used to interfere with intercellular signaling. However, current evidence in RA more strongly demonstrates the pathogenic effects of FLS-derived exosomes and the regulation of macrophage migration by microRNA-124-3p (miR-124-3p) than the establishment of mature hydrogel-based co-delivery systems [127]. Established FLS biology also indicates that blockade of a single cytokine is generally insufficient to eliminate the entire pathogenic network [128]. Studies in this area should therefore evaluate, at a minimum, macrophage states, FLS invasion, synovial thickness or pannus formation, and structural endpoints in cartilage and bone. Such evidence is required to support a conclusion of “crosstalk modulation” rather than general anti-inflammatory activity.

### 5.4. Adaptive Immune Tolerance: Potential and Evidentiary Boundaries

Dendritic cells (DCs), T cells, and B cells sustain autoimmune responses in RA, making antigen-specific tolerance an attractive long-term therapeutic objective. However, a substantial proportion of the available evidence is derived from studies of multiple sclerosis, general autoimmunity, or fundamental immune tolerance rather than RA hydrogel models [129,130,131,132]. Mechanisms involving programmed death-ligand 1 (PD-L1), indoleamine 2,3-dioxygenase (IDO), reduced expression of costimulatory molecules, and tolerogenic DCs may inform the design of material-based delivery systems. Nevertheless, this evidence is insufficient to conclude that intra-articular hydrogels can establish stable immune tolerance in RA.

**Figure 3:**
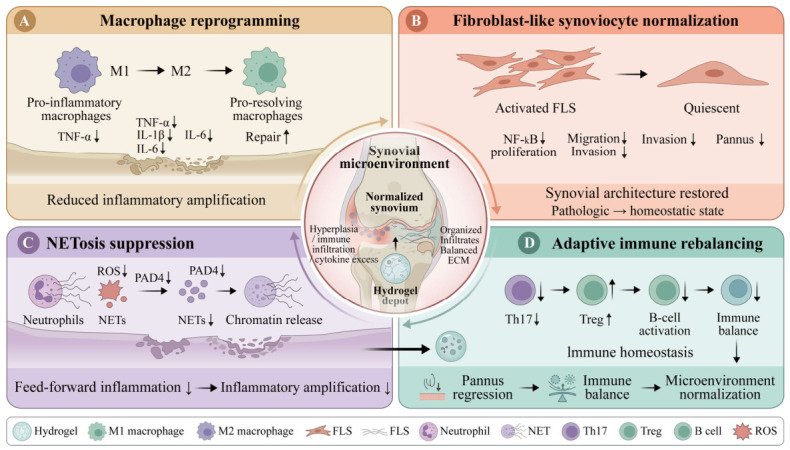
Synovial microenvironment remodeling by hydrogel-based local therapy in rheumatoid arthritis. Hydrogel depots may promote synovial normalization through coordinated regulation of macrophages, FLS, NETosis, and adaptive immune responses. (**A**) Pro-inflammatory macrophage states are attenuated, whereas inflammation-resolving phenotypes are enhanced, reducing TNF-α, IL-1β, and IL-6 production. (**B**) Activated FLSs acquire a less invasive phenotype, with decreased NF-κB activity, proliferation, migration, invasion, and pannus formation. (**C**) Suppression of ROS- and PAD4-associated NETosis reduces NET burden and feed-forward inflammatory amplification. (**D**) Potential adaptive immune rebalancing involves Th17 reduction, Treg enhancement, decreased B-cell activation, and restoration of immune balance. Together, these effects may convert the pathological synovial ecosystem into a more homeostatic microenvironment, although durable immune normalization remains to be demonstrated in RA-specific hydrogel studies.

Cooperative interactions between tolerogenic DCs and regulatory T cells (Tregs), type 1 regulatory T cells (Tr1 cells), or regulatory B cells (Bregs) have been supported in transplantation, allergy, and other immune models [133,134,135,136,137,138]. These findings may be used to formulate hypotheses for the local co-delivery of antigens, rapamycin, or immunomodulatory signals. Because RA is a systemic disease, it remains unclear whether tolerogenic signals delivered to a single joint can affect draining lymph nodes or systemic autoreactive clones. Nonspecific immunosuppression may also increase the risk of infection. The adaptive immune strategies described in this section should therefore be classified as “conceptual evidence.” Future studies must simultaneously demonstrate antigen specificity, the boundaries between local and systemic immune effects, the safety of repeated administration, and structural outcomes. Disease modification should not be inferred solely from changes in Treg proportions or cytokine levels. As illustrated in Figure 3, hydrogel-based interventions may remodel the RA synovial microenvironment by coordinating macrophage state regulation, FLS phenotype normalization, NETosis suppression, and adaptive immune rebalancing.

## 6. Cartilage Protection, Repair, and Boundaries of Regeneration

### 6.1. Antioxidant and MMP-Inhibitory Strategies and Chondrocyte Protection

Cartilage damage in RA is jointly driven by inflammation, oxidative stress, and matrix catabolism. Excessive ROS can damage chondrocyte mitochondria and amplify NF-κB–MMP signaling, whereas enzymes such as MMP-13 continuously degrade type II collagen and aggrecan [13,139]. At this stage, the principal value of hydrogels is to prolong local exposure, restrict systemic distribution, and reduce catabolic pressure through either the therapeutic cargo or the material itself. A crocin-loaded thermosensitive hydrogel reduced markers of oxidative stress and inflammation in an RA animal model [139]. Sustained-release studies involving antioxidants such as N-acetylcysteine and resveratrol provide additional transferable evidence for cartilage protection [140]. Components such as copper single-atom nanozymes with superoxide dismutase- or catalase-like activity may also confer sustained ROS-scavenging capacity on hydrogels. However, their therapeutic efficacy must still be validated in RA-specific models using structural endpoints [141].

Local inhibition of MMPs may reduce the off-target effects associated with systemic broad-spectrum inhibition. However, “reduced MMP expression” should be distinguished from “direct inhibition of matrix degradation.” An SPD-loaded ChSMA hydrogel reduced chondrocyte apoptosis and MMP expression in an RA-related coculture system, suggesting potential cartilage-protective activity [52]. Anti-inflammatory drug-loaded hydrogels may also indirectly reduce MMP activity by suppressing upstream cytokines [4,142]. Evaluation of MMP-inhibitory hydrogels should therefore include not only enzyme expression but also type II collagen, aggrecan, glycosaminoglycan content, and histological damage. This distinction is necessary to avoid equating improvements in molecular indicators directly with structural repair.

Hydrogels can also deliver exosomes, growth factors, or anti-inflammatory peptides to maintain chondrocyte viability and anabolic activity. Gelatin methacryloyl (GelMA) hydrogels loaded with bone marrow MSC-derived exosomes and KAFAK peptide delivery systems have shown the ability to regulate the inflammatory microenvironment and promote matrix production [143,144]. However, this evidence is derived primarily from OA, focal cartilage-defect, or general tissue-engineering models. It should therefore be regarded as transferable evidence rather than direct proof of structural regeneration in RA. Translation to RA will require further evaluation of stability in inflammatory synovial fluid, accessibility to the deeper cartilage layers, and long-term protective effects under persistent autoimmune pressure.

### 6.2. Restoration of Joint Lubrication and Reduction of Mechanical Wear

Alterations in HA, lubricin, and phospholipid composition in RA synovial fluid weaken boundary lubrication, causing inflammatory injury to be compounded by mechanical wear. Highly hydrated hydrogels can form a hydration layer on the cartilage surface and reduce contact stress and friction through viscoelastic cushioning [2,13]. However, the coefficient of friction is strongly influenced by the applied load, sliding velocity, counterface material, lubricating medium, and testing method. A single in vitro value should therefore not be used as a universal performance threshold for all intra-articular hydrogels.

Lubricin-derived domains, zwitterionic polymers, sulfonated HA, and chondroitin sulfate can enhance boundary lubrication through hydration repulsion and molecular-brush effects. The cartilage-binding peptide WYRGRL and dopamine groups can increase affinity for the cartilage surface and resistance to washout [2,13]. Most existing evidence is derived from in vitro friction tests, OA models, or cartilage-defect models. Their effectiveness in RA also depends on the proteolytic environment, synovial hyperplasia, synovial fluid turnover, and interfacial stability under repeated loading [84].

Improved lubrication should be regarded as a form of mechanical structural protection rather than as a surrogate for cartilage regeneration. A comprehensive evaluation should include in vitro friction and wear, intra-articular retention, cartilage-surface integrity, matrix loss, and weight-bearing behavior. A meaningful contribution to the structural progression of RA can be supported only when the lubricating effect is sustained and accompanied by preservation of cartilage structure.

### 6.3. Delivery of Stem Cells, Extracellular Vesicles, and Chondrogenic Factors

Hydrogels can improve the local retention and survival of MSCs and regulate chondrogenic differentiation through pore architecture, viscoelasticity, and growth-factor binding [145]. However, most evidence for “cell–scaffold–factor” systems is derived from OA or focal cartilage-defect models. The highly inflammatory, hypoxic, and protease-rich environment of RA joints may reduce cell survival and alter differentiation trajectories. RA applications must therefore demonstrate more than the upregulation of cartilage-associated markers. Cell fate, hypertrophic differentiation, immunocompatibility, and long-term integration with host cartilage should also be evaluated.

MSC-derived EVs can carry proteins, messenger RNA (mRNA), and miRNAs, and their sustained release from hydrogels may reduce rapid clearance [146]. Their lower cellular burden and amenability to engineering are potential advantages. However, properties such as “low immunogenicity, absence of tumorigenic risk, and ease of standardization” should not be assumed to be inherent. They remain dependent on the donor, culture conditions, isolation methods, and potency assays [147,148]. Engineered vesicles carrying microRNA-140 (miR-140) or signals associated with insulin-like growth factor 1 (IGF-1) have shown the ability to promote chondrocyte anabolism [149,150]. These findings provide a design rationale for RA applications, but direct RA models are still needed to determine whether these systems can simultaneously control synovitis and structural damage.

Chondrogenic signals such as transforming growth factor-β (TGF-β) and bone morphogenetic protein 7 (BMP-7) can promote the expression of SRY-box transcription factor 9 (SOX9) and matrix deposition [151,152]. However, sustained or excessive exposure may also induce chondrocyte hypertrophy, mineralization, or ectopic bone formation [153,154]. The role of hydrogels is therefore not merely to prolong release but to control the dose, spatial distribution, and sequence of exposure. Staged or responsive delivery may provide an engineering strategy for reducing the risk of hypertrophy [155,156]. At present, however, these approaches remain transferable tissue-engineering principles rather than established regenerative strategies in RA.

### 6.4. From Cartilage Protection to Functional Regeneration: A Conservative Evidence Hierarchy

To avoid overstating therapeutic efficacy, this review classifies cartilage-related outcomes into three levels. Cartilage protection refers to reductions in chondrocyte death, MMP activity, or matrix loss relative to controls. Structural preservation or repair additionally requires evidence that cartilage thickness, surface integrity, glycosaminoglycan content, or type II collagen is maintained or restored. Genuine functional regeneration should simultaneously demonstrate the formation of hyaline cartilage-like tissue, integration with adjacent cartilage and subchondral bone, mechanical behavior approaching that of native tissue, and sustained improvement in joint function. A single histological stain, increased cartilage thickness, or a reduced inflammation score is insufficient on its own to demonstrate regeneration.

According to these criteria, the triptolide-loaded SPT@TPL system, the IFX thermosensitive hydrogel, and the dexamethasone-loaded HA–Tyr hydrogel primarily achieve cartilage protection by reducing inflammation and matrix degradation [4,80,93]. Poly(ethylene glycol) dimethacrylate (PEGDMA) gradient scaffolds can provide defect filling and spatial support [157], whereas hierarchical MTX-loaded microcarriers reduce structural deterioration through immunomodulation [158]. In the absence of evidence for newly formed hyaline cartilage, interfacial integration, and functional testing, these outcomes are more appropriately classified as structural preservation or early repair rather than genuine regeneration.

Several RA studies have reported more pronounced repair-associated signals. The ChSMA@SPD system improved cartilage-matrix and bone-erosion indicators [52]. The HP@CEL supramolecular hydrogel improved ECM and bone structure by regulating macrophage–FLS crosstalk [18]. The TAP2 + Cx-HA system was associated with changes in cartilage thickness, glycosaminoglycan content, and new bone formation [95]. Collectively, these studies indicate that RA hydrogel research has advanced beyond simple anti-inflammatory treatment toward structural protection and preliminary repair. Nevertheless, high-quality, durable, and functionally complete cartilage regeneration remains to be demonstrated.

## 7. Control of Bone Erosion and Osteoimmune Modulation

### 7.1. Inhibition of Osteoclast Formation and Bone Resorption

Bone erosion in RA is driven primarily by sustained osteoclast differentiation and increased bone resorption within an inflammatory environment. Hydrogels offer material-based advantages over a single intra-articular injection by prolonging local exposure to anti-osteoclastic cargos, limiting systemic distribution, and modulating the pericellular microenvironment. However, anti-inflammatory activity, inhibition of osteoclast-mediated resorption, and new bone formation represent distinct levels of therapeutic efficacy. A reduction in RANKL expression or the number of TRAP-positive cells alone is insufficient to infer that bone erosions have been repaired.

A substantial proportion of the current evidence for anti-osteoclastic hydrogels is derived from models of periodontitis, osteoporosis, or general bone defects. RANKL-binding glycopeptide hydrogels, Drynaria flavonoids, and human β-defensin 3 hydrogels have all shown the ability to reduce inflammation- or osteoclast-related indicators [159,160,161]. Strontium-substituted hydroxyapatite and thermosensitive HA hydrogels delivering parathyroid hormone provide additional material-design rationales for regulating bone-cell responses [162,163]. Studies involving dendrobine, trifolirhizin, magnolol-containing composite hydrogels, and the circular RNA 28313/microRNA-195a/colony-stimulating factor 1 (circRNA_28313/miR-195a/CSF1) axis further suggest that osteoclast differentiation can be modulated through NFATc1, MAPK, oxidative-stress, or nucleic-acid regulatory pathways [164,165,166,167]. These findings constitute transferable evidence and cannot substitute for direct validation of bone structure in RA models.

In studies providing direct evidence in RA, budesonide-responsive hydrogels and all-active paeoniflorin hydrogels reduced osteoclast-related signaling or bone damage while suppressing inflammation [168,169,170]. RANKL/TNF-related inhibitory strategies, including the W9 peptide, also provide a rationale for selecting locally delivered cargos [170]. Future studies should combine cellular indicators, such as TRAP, CTSK, and NFATc1, with micro-computed tomography (micro-CT)-based bone erosion volume, trabecular parameters, and histological outcomes. Therapeutic efficacy should be elevated from an “anti-osteoclastic response” to “bone protection” only when preservation of bone structure has been demonstrated.

### 7.2. Regulation of the RANKL/OPG Axis and Inflammation–Osteoclast Coupling

Osteoclast activation in RA does not occur in isolation. TNF-α, IL-1β, IL-6, and interleukin-17 (IL-17) can increase RANKL expression in FLS and immune cells and cooperate with RANK signaling to activate the NF-κB, MAPK, and NFATc1 pathways [171,172,173,174]. NETs and their modified histones can also directly enhance RANKL-dependent osteoclastogenesis, thereby establishing a persistent feedback loop between synovial inflammation and bone erosion [175,176]. A hydrogel that delivers only a single antiresorptive agent without controlling upstream inflammation may therefore fail to produce stable structural benefits in bone.

Local hydrogels can simultaneously modulate inflammation and the RANKL/OPG balance. An enzyme-responsive budesonide hydrogel ameliorated adjuvant-induced arthritis by reducing inflammatory mediators [168]. The MTX- and Mg^2+^-loaded supramolecular hydrogel, ChSMA@SPD, and HPAP systems showed potential to suppress inflammation, reduce osteoclast differentiation, and protect bone or cartilage [7,52,101]. An HA–collagen interpenetrating-network hydrogel further combined bisphosphonate-functionalized components with zinc-doped calcium phosphate and simultaneously regulated macrophage, osteoclast, and osteogenic responses in an RA model [6]. By contrast, dual-factor OPG hydrogels and studies of the W9 peptide were conducted mainly in bone-defect or in vitro models and should be regarded as transferable design evidence for RA [169,170].

A single-point change in the RANKL/OPG ratio should not be equated with restoration of bone homeostasis. More comprehensive evidence should include inflammatory control, osteoclast number and activity, osteogenic markers, three-dimensional bone structure, and long-term recurrence. Multicargo systems should also define the release sequence, local concentration, and interactions of individual components to avoid antagonism among anti-inflammatory, anti-osteoclastic, and osteogenic signals.

### 7.3. Osteogenic Support and Repair of Bone Erosions

Hydrogels can support the repair of bone defects by providing shape-adaptable scaffolds, mineralizing components, and osteogenic signals. Bone morphogenetic protein 2 (BMP-2)-loaded chitosan hydrogels, self-assembling peptide nanofibers, and studies of substrate-stiffness regulation have demonstrated that both biochemical ligands and material mechanics can influence osteogenic differentiation of MSCs [177,178,179]. Recent stiffness-tuned phenolated hyaluronic acid/gelatin composite hydrogels further showed that Young’s moduli of approximately 3.3, 6.0, and 10.1 kPa differentially regulated human bone-marrow MSC adhesion, YAP localization, osteogenic marker expression, mineralization, and osteogenic cell-sheet fabrication, with the intermediate-stiffness hydrogel producing the strongest osteogenic response [180]. Studies of living joint prostheses incorporating BMP-2 and transforming growth factor-β3 (TGF-β3) have also demonstrated the engineering feasibility of osteochondral reconstruction [181]. However, these studies primarily addressed in vitro osteogenic cell-sheet engineering, cranial defects, general bone defects, or joint reconstruction and should be clearly treated as transferable design evidence rather than direct proof of RA-associated bone-erosion repair.

Repair of bone erosions in RA faces additional barriers, including persistent inflammation, sustained osteoclast activation, and insufficient local vascularization and cellular supply. Osteogenic cargos are therefore likely to exert stable effects only when inflammation and bone resorption are controlled concurrently. Interpenetrating-network hydrogels and self-healing injectable hydrogels have shown the potential to improve the osteoimmune environment and promote indicators associated with new bone formation in RA models [97]. Bone morphogenetic protein 9 (BMP9) can enhance osteogenic differentiation of RA-associated MSCs, but this finding does not yet constitute direct evidence of hydrogel-mediated repair [182]. Collectively, these findings support the further development of osteogenic systems but cannot be extrapolated to claim that regeneration of bone erosions has already been achieved.

Evidence of bone repair should extend beyond the upregulation of osteogenic genes. In addition to runt-related transcription factor 2 (RUNX2), alkaline phosphatase (ALP), osteocalcin (OCN), and mineralization staining, studies should determine whether erosion defects are filled with new bone, whether trabecular architecture is reconstructed, whether newly formed bone integrates with host tissue, and whether these changes persist after inflammatory relapse or repeated loading. Sequential release involving “initial control of inflammation and osteoclast activity, followed by osteogenic stimulation” represents a reasonable design hypothesis. However, the relevant temporal window should be determined by disease status and pharmacokinetics rather than prescribed as a fixed period.

### 7.4. Remodeling the Osteoimmune Microenvironment of Bone-Erosion Sites

Osteoimmune modulation emphasizes the coupling among immune cells, FLS, osteoclasts, and osteoblasts rather than the simple addition of anti-inflammatory and osteogenic functions. Within the bone-erosion niche in RA, RANKL/OPG imbalance, inflammatory cytokines, and NETs collectively promote osteoclastogenesis and suppress effective bone formation [171,172,173]. An ideal hydrogel should therefore simultaneously account for the resolution of inflammation, restriction of osteoclast activity, support of osteogenesis, and restoration of local homeostasis after material degradation.

A bone-targeted MTX–alendronate conjugate reduced inflammation and bone loss in a CIA model, demonstrating the value of bone-surface targeting, although the system itself was not a hydrogel [183]. By contrast, a self-healing injectable hydrogel regulated macrophages, inhibited osteoclast activity, and supported bone repair in an RA model, providing more direct evidence for an osteoimmune biomaterial strategy [97]. The BMP9 study further provides a mechanistic basis for osteogenic signaling under RA conditions [182]. These findings suggest that bone-affinitive groups, immunomodulatory cargos, and mineralizing scaffolds can be combined. Nevertheless, the independent contribution of each functional module must be resolved through appropriate control experiments.

In summary, bone-related efficacy of RA hydrogels should be classified into four levels: improvement in molecular or cellular indicators of osteoclast activity, inhibition of bone-erosion progression, new bone formation within the defect, and stable repair accompanied by restoration of mechanical and joint function. Current evidence provides relatively strong support for the first two levels. Evidence for new bone formation is derived mainly from a limited number of preclinical studies, whereas functional bone repair remains insufficiently demonstrated. Clearly defining these evidentiary boundaries is more consistent with the requirements for evaluating structural disease modification than broadly claiming “bone regeneration.”

## 8. Multitissue Coverage and Adaptation to Pathological States

**Figure 4:**
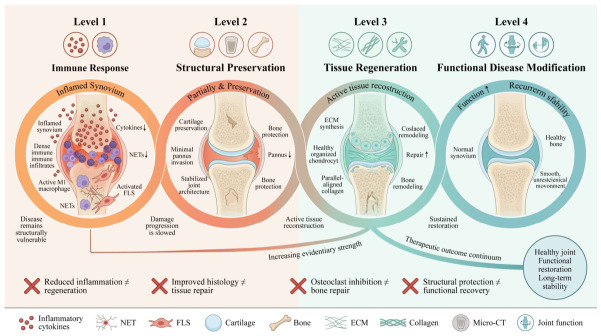
Evidence hierarchy for structural disease modification in rheumatoid arthritis hydrogel studies. Hydrogel efficacy in rheumatoid arthritis should be assessed across four escalating evidence stages. **Level 1** indicates local immune response modulation, including reduced cytokines, immune-cell infiltration, activated macrophage states, FLS activation, and NET burden, but does not prove structural repair. **Level 2** indicates structural preservation, including cartilage protection, reduced pannus invasion, stabilized joint architecture, bone protection, and slowed damage progression. **Level 3** requires tissue repair or regeneration-level evidence, including extracellular matrix synthesis, organized chondrocytes, collagen network restoration, bone remodeling, and host-tissue integration. **Level 4** represents functional disease modification, requiring sustained structural restoration, normal synovial architecture, healthy bone, smooth joint motion, functional recovery, and long-term stability. The framework highlights that inflammation reduction, histological improvement, osteoclast inhibition, and structural improvement should not be equated with regeneration or functional recovery.

Synovial inflammation, cartilage-matrix degradation, and bone erosion are pathologically coupled in RA joints, but their relative predominance varies among patients and across disease stages. The central objective of multitissue treatment is therefore not simply to increase the number of cargos. Instead, material functions should be configured according to the pathological compartment, tissue accessibility, and therapeutic objective, and their actual coverage should be validated using corresponding structural endpoints. To avoid overinterpreting short-term anti-inflammatory or histological findings, we propose a hierarchical evidence framework for evaluating hydrogel-mediated structural disease modification in RA, as summarized in Figure 4.

### 8.1. From Single-Compartment Intervention to Multitissue Coverage

Most hydrogel studies in RA began with local drug delivery to the synovium. A dexamethasone-loaded HA–Tyr hydrogel prolonged intra-articular exposure and reduced inflammatory mediators, but its principal evidence remained focused on the synovium and short-term histological improvement [4]. Subsequently developed adhesive lubricating hydrogels and nanocomposite hydrogels combined anti-inflammatory activity with mechanical protection or cartilage-related outcomes, thereby extending the therapeutic scope from the synovium to cartilage [13,16]. Systems such as ChSMA@SPD and Gel-MTX/Mg further reported cartilage preservation, inhibition of osteoclast activity, or improvement in bone structure, suggesting that hydrogels can affect multiple pathological compartments [7,52]. However, these findings are still derived mainly from single-administration protocols and short-term animal experiments. Recent reviews of intra-articular hydrogels likewise indicate that only a small proportion of studies have simultaneously quantified synovial, cartilage, bone, and functional endpoints [184].

The classification of a system as single-compartment, dual-compartment, or multicompartment should therefore be based not merely on cargo design but on the tissue-specific endpoints that were actually measured. Reduced inflammatory mediator levels do not automatically demonstrate cartilage benefit, and improved cartilage staining cannot substitute for evaluation of bone erosion. Structural damage in RA involves pannus invasion, cartilage-matrix loss, and osteoclast-mediated bone destruction [185,186,187,188]. A multitissue system should be considered to have the potential for multitissue structural modification only when direct evidence is obtained in each relevant compartment. To avoid inferring multitissue efficacy from a single inflammatory or histological indicator, Table 3 summarizes the endpoints actually assessed in representative RA hydrogel studies across four dimensions—synovium, cartilage, bone, and function—and defines their evidentiary boundaries. As shown in Table 2, most studies demonstrate attenuation of synovial inflammation, and some systems further show cartilage preservation or inhibition of bone erosion. However, studies that simultaneously cover the synovium, cartilage, bone, and direct endpoints of joint function remain limited.

### 8.2. Prioritizing Treatment According to Pathological State

The conventional categories of “early,” “progressive,” and “late-stage” disease can help organize the discussion, but they should not be interpreted as a fixed, linear therapeutic timeline. Early RA is generally regarded as a window of opportunity for improving long-term outcomes [189,190,191]. However, imaging modalities such as magnetic resonance imaging (MRI) can detect subclinical synovitis, bone marrow edema, or early erosions during the clinically early phase [192]. This review therefore prioritizes treatment according to the current pathological state rather than a fixed number of weeks.

When inflammatory activity predominates and there is no clear evidence of extensive structural defects, material design should prioritize rapid local action, appropriate retention, and anti-inflammatory or antioxidant release that can terminate when no longer required. Bioresponsive delivery systems provide a general design rationale for modulating release according to inflammatory signals [193]. MTX liposomal hydrogels, NO-scavenging nanogels, and dexamethasone-loaded thermosensitive hydrogels have respectively demonstrated preclinical value in local drug delivery, regulation of oxidative/nitrosative stress, and control of pain and inflammation [194,195,196]. When cartilage catabolism and impaired lubrication become the principal risks, additional functions such as MMP regulation, adhesion to the cartilage surface, or mechanical lubrication should be incorporated. An in situ hydrogel co-delivering an anti-inflammatory drug and MMP-9 siRNA, together with a leflunomide-loaded lipid nanocarrier hydrogel, produced cartilage-related improvements. However, the extent of “repair” must be interpreted cautiously on the basis of newly formed matrix and long-term functional endpoints [92,197].

When synovial invasion, persistent cartilage loss, and bone erosion occur concurrently, simply increasing the anti-inflammatory dose is insufficient to address the full spectrum of structural risks. FLS invasiveness, inflammation–osteoclast coupling, and RANKL-associated bone damage represent important therapeutic targets in this pathological state [186,187]. miRNA-mediated regulation of synovium–bone signaling and inhibition of RANKL provide mechanistic rationales for local multitarget designs [198,199]. However, incorporating an FLS inhibitor, a cartilage growth factor, and an anti-osteoclastic cargo into the same hydrogel remains largely an unvalidated strategy and cannot be taken as evidence that synchronous repair has been achieved. RA pathology and therapeutic responses are highly heterogeneous [188,200], and cargo selection should therefore be jointly guided by tissue-specific endpoints and pharmacokinetics.

**Figure 5:**
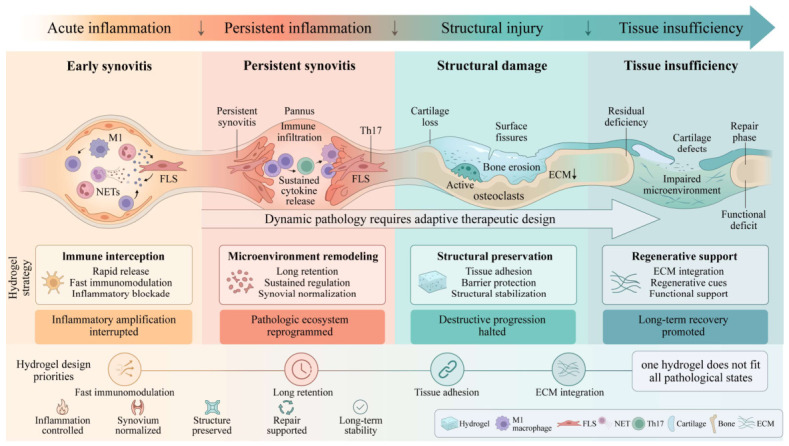
Stage-adaptive hydrogel strategies across rheumatoid arthritis progression. RA pathology progresses from early inflammatory synovitis and persistent inflammatory arthritis to structural deterioration and post-inflammatory tissue insufficiency. Hydrogel design should therefore be adapted to the dominant pathological state. Early inflammatory synovitis requires immune interception through rapid release, fast immunomodulation, and inflammatory blockade. Persistent inflammatory arthritis requires long retention, sustained regulation, and synovial microenvironment remodeling. Structural deterioration requires tissue adhesion, barrier protection, and structural stabilization to preserve cartilage and bone. Post-inflammatory tissue insufficiency requires regenerative support through ECM integration, regenerative cues, and functional support. This framework emphasizes that “one hydrogel does not fit all pathological states” and that hydrogel design priorities should shift from inflammation control to synovial normalization, structural preservation, repair support, and long-term stability.

When systemic and local inflammation have been relatively controlled but focal osteochondral defects persist, hydrogels may be more appropriately used as cellular scaffolds or regenerative microenvironments. Composite hydrogels incorporating engineered chondrocalcin or carbon quantum dots suggest the feasibility of promoting chondrogenic differentiation [201,202]. However, the former remains an emerging approach, whereas the latter primarily provides general evidence from cartilage engineering. Neither can be taken as direct evidence of the clinical reparability of late-stage RA joints. In severe deformity or extensive full-thickness defects, hydrogels are more likely to serve as adjuncts to systemic therapy and surgical reconstruction than as substitutes for surgery. As illustrated in Figure 5, the dominant therapeutic objective of hydrogel-based intervention should shift with the pathological stage of RA, from immune interception in early synovitis to microenvironment remodeling, structural preservation, and regenerative support in later disease states.

### 8.3. Spatial and Sequential Release: Design Rationale and Validation Requirements

The sequence of “first control inflammation, then protect tissue, and finally promote repair” has a reasonable biological basis. However, RA does not have universally applicable fixed windows of 0–2 weeks, 2–6 weeks, or 6–12 weeks across all models and patients. A more reliable sequential-release strategy should be triggered by measurable pathological signals, such as ROS, complement activation, or protease activity during the inflammatory peak, followed by signals associated with matrix stabilization and cell recruitment. Exosome-inspired light-triggered gels and oligonucleotide hydrogel microspheres have demonstrated the possibility of using material architecture to intervene in immune feedback loops [203,204]. Whether these systems truly generate temporally distinct multitissue benefits must still be validated through intra-articular pharmacokinetics and serial histological analyses.

Future sequential or spatially compartmentalized hydrogels should address at least three questions. First, do individual cargos reach their intended tissues rather than remaining only in the synovial fluid? Second, is the release sequence confirmed by in vivo concentration–time data? Third, do subsequent reparative signals act only after inflammation has been adequately controlled? “Temporally programmed therapy” can be transformed from a conceptual model into a verifiable therapeutic strategy only when the spatial localization of the material, release kinetics, and synovium–cartilage–bone endpoints are aligned.

## 9. Evidence Evaluation and Clinical Translation

### 9.1. From Endpoint Listing to a Study-Level Four-Dimensional Evidence Matrix

The purpose of the four-dimensional evidence matrix is not to increase the number of measurements, but to map each study onto four interconnected dimensions: the synovium, cartilage, bone, and function. Synovial assessment should integrate histology, imaging, and cellular networks rather than report cytokines alone. Studies correlating MRI with pathological findings support the combined interpretation of synovial thickness, pannus formation, and bone marrow changes [205]. Inflammatory mediators such as interleukin-32 (IL-32) may serve as mechanistic biomarkers, but a reduction in a single biomarker does not indicate structural improvement [206]. Cartilage and bone assessments should respectively include matrix preservation and quantitative evaluation of bone erosion while also accounting for cross-compartment signals such as cartilage-derived RANKL [207]. Functional assessment should include gait, weight bearing, range of motion, or pain-related behavior and should establish associations with structural changes. Exercise-intervention studies have shown that changes in the synovium, cartilage, and subchondral bone may occur asynchronously, further demonstrating that a single endpoint cannot represent overall therapeutic benefit [208].

### 9.2. Immune Response, Structural Protection, Tissue Repair, and Functional Structural Modification

Existing studies can be classified into four levels according to the endpoints reported. Level I comprises immune responses, including improvements in inflammatory mediators, immune-cell states, or joint swelling. Level II comprises structural protection, defined as less cartilage or bone damage than in the control group without evidence of newly formed tissue. Level III comprises tissue repair and requires defect filling and the formation of new matrix or new bone. Level IV comprises functional structural modification and requires sustained structural benefits accompanied by recovery of joint function. This classification should be based on the actual findings rather than on the authors’ use of terms such as “regeneration” or “disease modification.” On the basis of these criteria, Table 3 further stratifies representative hydrogel studies with direct in vivo evidence in RA according to model characteristics, administration route, observation design, structural and functional endpoints, and level of evidence. As shown in Table 4, current studies are concentrated primarily at Level I, immune response, and Level II, structural protection. A small number of studies using focal bone or osteochondral defect models reach Level III, tissue repair, but no system has yet adequately satisfied all criteria for Level IV, functional structural modification.

For example, MTX liposomal hydrogels and leflunomide-loaded nanocarrier hydrogels reported both inflammatory and partial histological improvements and are therefore more appropriately provisionally classified as providing structural protection rather than solely an immune response or proven regeneration [185,188]. Inflammation-responsive microspheres and D-amino acid-based supramolecular hydrogels primarily support on-demand release, synovial control, and structural preservation [209,210]. IFX hydrogels likewise primarily demonstrate cartilage protection [93]. Systems such as CeNZs/KGN@P407 show potential for bone or cartilage repair, but evidence regarding the quality of newly formed tissue, interfacial integration, and long-term mechanical performance is still required before they can be assigned to Level III [55]. T follicular helper (Tfh) cell-regulating hydrogel microneedles primarily provide evidence of immunomodulation and should not be classified as cartilage regeneration solely because arthritis is alleviated [211]. Similarly, although Gel-MTX/Mg, sinomenine-loaded hydrogels, and dual-drug supramolecular hydrogels improve disease scores and structural indicators, they cannot be classified as Level IV functional structural modification in the absence of direct functional measurements such as gait, weight bearing, or range of motion [5,7,53].

**Table 4:** Representative Hydrogel Studies with Direct In Vivo Evidence in Rheumatoid Arthritis: Model Design, Evaluation Endpoints, and Levels of Evidence.

Hydrogel System	RA Model and Model Characteristics	Administration Route and Observation Design	Principal In Vivo Evaluation Endpoints	Highest Level of Evidence	Evidentiary Boundaries and Major Limitations	Reference
Dexamethasone-loaded HA–Tyr hydrogel	Collagen-induced arthritis (CIA) model; primarily recapitulates immune-mediated synovitis and secondary joint damage	Intra-articular injection; short-term therapeutic effects following sustained local release were evaluated	IL-6, PGE2, and multiple cytokines; H&E staining of joint tissues	Level I: Immune response	Evidence was based mainly on reductions in inflammatory mediators and improvement in overall histopathology; the cartilage matrix, bone erosion, and joint function were not separately quantified, precluding a conclusion of structural modification	[4]
MTX-loaded click-crosslinked Cx-HA drug depot	RA rat model; used to evaluate intra-articular drug retention and local treatment	Single intra-articular injection; free MTX, MTX-HA, and MTX-Cx-HA were compared	Intra-articular drug distribution, arthritis index, cartilage thickness, chondrocytes and glycosaminoglycan deposition, inflammatory mediators, and histological indicators related to new bone formation	Level III: Signals of tissue repair	Chondrocytes, glycosaminoglycan deposition, and new bone formation were reported, but the mechanical properties of the newly formed tissue, interfacial integration, and standardized joint-function assessments were lacking; histological repair cannot be directly equated with mature functional regeneration	[212]
IFX-loaded F127–HA–PGA thermosensitive hydrogel	Ovalbumin/complete Freund’s adjuvant (OVA/CFA)-induced rabbit knee arthritis model; suitable for evaluating intra-articular cartilage and pain-related behavior in a relatively large joint	A single 0.5 mL dose was injected into the affected knee after model establishment; follow-up lasted 6 weeks	Joint temperature and diameter, inflammatory mediators in synovial fluid, synovial histology, gross cartilage examination, H&E, toluidine blue, and Safranin O staining, COL I/COL II, weight-bearing index, and paw-withdrawal threshold	Level II: Structural protection with pain and weight-bearing benefits	Clear evidence of cartilage protection and behavioral benefit was obtained, but bone erosion was not evaluated; a 6-week observation period is insufficient to demonstrate durable structural modification, and pain relief does not indicate tissue regeneration	[93]
IND/MTX/MMP-9 siRNA in situ hydrogel	Mouse arthritis model; simultaneously targets inflammation and MMP-9-mediated matrix degradation	Intra-articular injection of the composite nanogel; compared with formulations containing individual or partial cargos	Paw and ankle swelling, TNF-α, IL-6, and MMP-9, ankle morphological parameters, and histology	Level II: Structural protection	The ankle morphology approached normal and suggested reduced cartilage injury, but the formation, integration, and mechanical properties of newly formed hyaline cartilage were not demonstrated; direct functional endpoints were lacking	[92]
DNase I-functionalized dynamic hydrogel	CIA mouse model; focuses on inflammatory amplification driven by the persistent presence of NETs	Intra-articular injection of the DNase-functionalized hydrogel; combined with MTX in some experiments	NET- and citrullinated histone-related indicators, inflammatory mediators, arthritis scores, paw swelling, and joint histology	Level I: Immune response	Direct evidence showed that sustained NET degradation alleviated inflammation, but cartilage, bone, and functional endpoints were incomplete; general histological improvement is insufficient to elevate the evidence to structural modification	[73]
TAP2-loaded click-crosslinked HA hydrogel	RA animal model; uses Toll-like receptor 4 (TLR4)-associated innate immune signaling to model persistent synovitis and structural damage	Intra-articular injection; free peptide, non-crosslinked carrier, and click-crosslinked hydrogel were compared	In vivo peptide stability and retention, arthritis index, inflammatory mediators, cartilage thickness, glycosaminoglycans, and bone-related histology	Level II: Structural protection	Supports cartilage-matrix preservation and shows signals associated with tissue repair, but evidence of interfacial integration of newly formed tissue, mechanical testing, and direct functional evaluation was lacking, preventing classification as mature Level III tissue repair	[95]
HP@CEL HA–nanodrug supramolecular hydrogel	RA rodent model; focuses on macrophage–FLS crosstalk	Local intra-articular administration; free drug, nanocarrier, and composite hydrogel were compared	Joint swelling and arthritis scores, macrophage phenotypes, FLS activation, inflammatory mediators, cartilage ECM, and histological indicators related to bone structure	Level II: Multitissue structural protection	Synovial, cartilage, and bone-related indicators were assessed, but cartilage and bone outcomes relied mainly on histology; long-term micro-CT, tissue mechanics, and functional endpoints were lacking	[18]
SPT@TPL dual dynamically crosslinked hydrogel	RA rodent model; oxidative stress and the inflammatory microenvironment serve as the principal therapeutic targets	Intra-articular injection; the synergistic effects of material-mediated ROS regulation and sustained triptolide release were evaluated	Paw swelling, arthritis scores, inflammatory mediators, ROS, macrophage states, and articular-cartilage histology	Level II: Structural protection	Improvements in cartilage staining and surface morphology support a protective effect, but the formation of new hyaline cartilage, integration with subchondral bone, and restoration of joint function were not demonstrated	[80]
Anti-inflammatory–osteogenic HA/collagen interpenetrating-network hydrogel	RA bone-erosion microenvironment model incorporating a focal bone-erosion/defect repair setting	The injectable hydrogel was adapted to the bone-erosion region; staged anti-inflammatory, anti-osteoclastic, and osteogenic effects were evaluated	Macrophage states, TRAP and osteoclast-related indicators, osteogenic markers, micro-CT-derived bone-volume fraction and trabecular parameters, new bone formation, and tissue integration	Level III: Bone tissue repair	Provides relatively direct evidence of new bone formation and bone-defect filling; however, an experimentally created focal defect differs from naturally progressive marginal erosion in RA, and recovery of overall joint mechanics or motor function was not demonstrated	[6]
DNRS dual-gas-regulating self-healing hydrogel	RA animal model characterized by excess NO, insufficient H_2_S, macrophage dysregulation, and osteoclast activation	Intra-articular injection; the hydrogel scavenged NO and released H_2_S and MTX under pathological conditions	Joint swelling and scores, inflammatory mediators, macrophage states, TRAP, bone histology, and micro-CT bone parameters	Level II: Structural protection with signals of bone repair	Demonstrated suppression of inflammation and osteoclast activity together with improved bone microstructure, but stable filling with newly formed bone and long-term integration with host tissue were not adequately established	[97]
DAGQD@Cu@KGN–SO_3_^−^/DA-HA adhesive lubricating hydrogel	Two-stage model: CIA rats at an early stage and OIA rabbits with superimposed standardized full-thickness osteochondral defects at a later stage	CIA rats received intra-articular administration on the day of the second immunization and were observed for 4 weeks; OIA rabbits were observed for 8 weeks after treatment of the defect site	Rats: arthritis scores, micro-CT, cartilage histology, SOX9, type II collagen (COL II), aggrecan (ACAN), and inflammatory mediators; rabbits: gross defect examination, micro-CT, HSS/Osteoarthritis Research Society International (OARSI) scores, bone volume/tissue volume (BV/TV), trabecular number (Tb.N), and indentation modulus of newly formed tissue	Level III: Cartilage/osteochondral tissue repair	This is one of the systems with the most comprehensive structural assessments and includes local mechanical testing of newly formed tissue; however, the later-stage model combined an inflammatory background with an artificial defect, and Level IV functional structural modification was not demonstrated by long-term gait, weight-bearing, or range-of-motion assessments	[13]
CuS-T/ChSMA MMP-9-binding hydrogel	Adjuvant-induced arthritis (AIA) mouse model; emphasizes synovial pannus and MMP-9-mediated cartilage injury	Intra-articular injection of a photocrosslinked hydrogel; non-targeted CuS and MMP-9-binding CuS-T were compared	Arthritic symptoms, synovial inflammation, RA-FLS invasion, macrophage states, MMP-9, MAPK signaling, COL II, aggrecan, and cartilage histology	Level II: Synovium–cartilage dual-compartment structural protection	Demonstrated an association between local MMP-9 binding and improvement in the cartilage matrix, but mature cartilage-defect filling, interfacial integration, cartilage mechanics, and bone-erosion assessment were lacking	[72]
Polymer-modified DNA hydrogel co-delivering functional mitochondria and Prussian blue nanozymes	RA animal model; primarily targets intracellular and extracellular oxidative stress and mitochondrial dysfunction	Intra-articular injection of a composite DNA hydrogel containing functional mitochondria and nanozymes	ROS and mitochondrial function, inflammatory mediators, macrophage and synovial states, and cartilage and osteochondral histology	Level II: Multitissue structural protection	Cartilage- and bone-related repair signals were observed, but the in vivo survival, tissue localization, and long-term effects of the functional organelles remain unclear; standardized functional assessments and long-term recurrence monitoring were lacking	[16]
ChSMA@SPD spermidine hydrogel	CIA mouse model supplemented by an RA patient-derived synovial organoid–chondrocyte coculture model	Intra-articular administration; eight animals per group were used in the CIA in vivo experiments	Arthritis scores and incidence, paw-pad thickness, H&E and Safranin O staining, COL2A, MMP3, macrophage states, micro-CT, BV/TV, trabecular thickness (Tb.Th), and trabecular separation (Tb.Sp)	Level II: Cartilage and bone structural protection	Provides evidence of both cartilage preservation and bone structural parameters, but the in vivo bone findings mainly indicate reduced erosion or structural preservation; integrated new bone formation within erosion sites was not demonstrated, and direct functional endpoints were not included	[52]
Gel-MTX/Mg supramolecular hydrogel	RA rat model; focuses on inflammation and osteochondral destruction	Single intra-articular administration; free MTX, individual components, and the complete Gel-MTX/Mg formulation were compared	Paw swelling and arthritis scores, inflammatory mediators, cartilage histology, osteoclast-related indicators, and bone microstructure	Level II: Multitissue structural protection	A single administration improved inflammatory and osteochondral indicators, but the evidence primarily supports structural preservation rather than new tissue formation within defect sites; standardized gait, weight-bearing, and range-of-motion assessments were absent	[7]

### 9.3. Model Extrapolation, Intra-Articular Fate, and Long-Term Safety

Collagen-induced arthritis (CIA), adjuvant-induced arthritis (AIA), and cytokine-driven models can reproduce selected immune and structural phenotypes of RA. However, their disease course, joint size, load distribution, and systemic immune background differ from those of human RA [200]. Models driven by a single cytokine also cannot adequately represent multipathway regulation involving tumor necrosis factor receptor 1 (TNFR1), tumor necrosis factor receptor 2 (TNFR2), and other pathways or the heterogeneity among patients [213]. Model selection should therefore be guided by the intended application. Studies of local drug delivery may prioritize inflammation and pharmacokinetics, whereas studies of tissue repair require longer follow-up, larger joints, and models capable of generating stable defects. Existing reviews of injectable delivery systems indicate that most evidence remains limited to small-animal models and short-term observations [184,214].

The intra-articular fate of a hydrogel should encompass material retention, cargo release, tissue penetration, lymphatic and systemic clearance, and degradation-product disposition. In vitro release profiles cannot substitute for in vivo pharmacokinetic analyses because synovial-fluid turnover, enzymatic activity, inflammatory exudate, joint motion, and particle uptake can substantially alter release and clearance. Nanocrystals, nanocarriers, and composite hydrogels additionally require independent assessment of particle migration, cellular uptake, synovial accumulation, lymph-node drainage, and plasma exposure [215]. Repeated-injection safety should be tested directly rather than inferred from single-dose cytocompatibility. Essential endpoints include synovial foreign-body reaction, fibrosis, infection risk, local mechanical interference, cartilage-surface wear, systemic exposure, and persistence of degradation products. Non-RA biomechanical and material-durability studies can inform the test design but should not be cited as direct evidence of RA efficacy [216,217].

### 9.4. Manufacturing, Sterilization, Quality Control, and Clinical Positioning

Clinical translation requires the establishment of critical quality attributes (CQAs) appropriate to the product configuration. These attributes include the molecular weight and degree of substitution of the raw materials, degree of crosslinking, rheological behavior, gelation window, cargo content, release profile, degradation products, and endotoxin level. Studies of nanostructured lipid carriers and MTX-loaded thermosensitive hydrogels demonstrate that particle size, dispersibility, encapsulation efficiency, and the sol–gel transition can affect drug exposure and therapeutic efficacy [218,219]. Intra-articular dual-drug hydrogels further indicate that co-loaded drugs may alter gel formation and release kinetics [91,220]. Systems containing TNF inhibitors, nonsteroidal anti-inflammatory drugs (NSAIDs), or self-assembled natural products also require validation of preserved biological activity, storage stability, and batch-to-batch consistency [221,222,223].

Sterilization strategies cannot be applied uniformly across hydrogel systems. Terminal irradiation, moist-heat sterilization, or ethylene oxide treatment may alter polymer chains, crosslinked networks, or protein activity. Filtration through a 0.22 μm membrane is applicable only to filterable, low-viscosity precursors or solutions and cannot be used for preformed gels, cellular formulations, or all extracellular-vesicle products. Systems that cannot undergo terminal sterilization should be manufactured using sterile raw materials, aseptic compounding, and aseptic filling, and the effects of these processes on material performance should be validated. The sterility assurance level should be expressed as SAL 10^−6^, rather than “<10^−6^ colony-forming units per gram (CFU/g).” In addition, gelation time, swelling, and modulus should be specified according to the intended use, such as a drug depot, lubricating layer, or tissue scaffold, rather than assigned uniform thresholds.

Local hydrogel therapy should be positioned as an adjunct to conventional synthetic, biologic, or targeted synthetic DMARDs rather than as a replacement for systemic treat-to-target management. Plausible applications include persistent activity in one or a small number of joints despite otherwise adequate systemic therapy, the need for prolonged local drug exposure, focal cartilage-surface protection, local inhibition of erosion progression, or repair support for a defined residual defect when inflammatory activity is adequately controlled. Claims of systemic dose reduction, prevention of radiographic progression, or disease modification require combination-therapy studies that assess local and systemic pharmacokinetics, systemic disease activity, infection-related safety, structural imaging, and functional outcomes.

## 10. Conclusions and Perspectives

Current evidence supports hydrogels primarily as local delivery and microenvironment-modulating platforms for RA. Their most consistently demonstrated benefits are prolonged local exposure and attenuation of synovial inflammatory activity. Selected systems additionally preserve cartilage matrix or reduce bone erosion, and a small number of focal-defect studies provide repair-associated evidence. However, reductions in cytokines, improved cartilage staining, fewer osteoclasts, or short-term histological improvement should not be described as regeneration or structural disease modification. These claims require new tissue formation, organized matrix, host integration, mechanical competence, and durable functional recovery.

Hydrogel requirements should be matched to the intended clinical function. Intra-articular depots require validated injectability, retention, cargo stability, pharmacokinetics, degradation, and repeat-dose safety. Cartilage-surface systems additionally require wet adhesion, lubrication, wear resistance, and structural protection under cyclic loading. Repair-oriented scaffolds require new tissue formation, integration, remodeling, and mechanical validation. Increasing the number of cargos or responsive modules does not by itself increase the level of therapeutic evidence.

Future studies should integrate RA-relevant long-term models, tissue-specific synovial–cartilage–bone endpoints, in vivo concentration–time and biodistribution data, repeated-administration safety, and validated functional outcomes. Manufacturing studies should define critical quality attributes and assess the effects of sterilization or aseptic processing on the final product. Clinically, hydrogel therapy should be developed as an adjunct to systemic DMARD treatment for selected joints with persistent local activity, a need for prolonged local exposure, focal structural protection, or a defined repair requirement. Until durable multitissue benefit and functional recovery are demonstrated, RA hydrogels should be described as local structural-protective platforms with disease-modifying potential, not as established disease-modifying therapies.

## Figures and Tables

**Table 1:** Operational framework for evidence classification and outcome grading.

Evidence or Outcome Level	Operational Definition Used in This Review	Examples of Acceptable Endpoints	Interpretation Allowed
Direct RA evidence	Evidence generated using human RA-derived material, clinical RA samples, or established RA-relevant experimental inflammatory arthritis models	CIA, AIA, K/BxN or RA-FLS; synovitis, pannus, cartilage matrix, erosion, gait.	Supports RA-relevant efficacy within the tested model, route, and endpoint
Transferable evidence	OA, bone-defect, wound-healing, inflammation, or tissue-engineering model used to support a material-design principle.	Lubrication, stiffness-tuned osteogenesis, generic ROS/MMP-responsive release.	Supports design rationale only; requires RA validation.
Conceptual evidence	Mechanistic or materials-science data without disease-specific validation.	Crosslinking chemistry, in vitro release trigger, isolated cell mechanism.	Hypothesis-generating; not proof of RA efficacy.
Level I: local immunomodulation	Reduction in inflammatory activity without proven structural benefit.	Cytokines, macrophage/FLS markers, paw swelling, arthritis score.	Anti-inflammatory or immunomodulatory activity.
Level II: structural protection	Less cartilage or bone damage than controls but no confirmed new tissue formation.	Safranin O, COL2A/aggrecan, TRAP, micro-CT erosion metrics.	Structural protection or anti-erosive effect.
Level III: tissue repair	Defect filling or new matrix/bone formation with host-tissue integration assessed.	New cartilage/bone, matrix organization, integration, remodeling.	Repair-associated evidence; not automatically functional disease modification.
Level IV: functionally validated structural disease modification	Durable multitissue structural benefit plus functional recovery.	Long-term imaging/histology plus gait, weight bearing, range of motion, pain behavior.	Highest preclinical evidence for disease-modifying potential.

**Table 2:** Representative Local Hydrogel Platforms Supported by Direct Evidence in Rheumatoid Arthritis: Material Composition, Gelation Mechanisms, and Delivery Characteristics.

Hydrogel System	Hydrogel Matrix and Gelation Method	Cargo or Functional Component	Primary Delivery/Response Mechanism	Study Model and Administration Route	Main Translational Limitation/Evidentiary Boundary	Reference
HA–Tyr hydrogel	Tyramine-modified hyaluronic acid; horseradish peroxidase/hydrogen peroxide (HRP/H_2_O_2_)-catalyzed oxidative coupling of phenolic groups to form a covalent network	Dexamethasone	The gel network restricts drug diffusion and prolongs local exposure	CIA animal model; intra-articular injection	evaluation focused mainly on inflammation and short-term histological changes, without establishing complete intra-articular pharmacokinetics	[4]
Cx-SIS drug depot	Tetrazine-modified and trans-cyclooctene-modified small intestinal submucosa (SIS) matrix rapidly crosslinked through a bioorthogonal click reaction	Methotrexate (MTX)	Gelation occurs within seconds after injection; the extracellular matrix (ECM) network improves intra-articular retention and enables sustained MTX release	RA animal model; intra-articular injection	histological findings indicated preservation of cartilage and glycosaminoglycans, but these findings should not be directly interpreted as functional evidence	[88]
FK506 nanoparticle–thermosensitive hydrogel	Self-assembled tacrolimus nanoparticles incorporated into a thermosensitive polymer hydrogel	Tacrolimus	The nanocarrier and thermosensitive gel form a secondary delivery system that reduces rapid diffusion	RA animal model; local intra-articular administration	the long-term intra-articular fates of the gel, nanoparticles, and free drug were not systematically compared	[89]
Electrostatic-interaction drug depot	A carboxyl-containing mPEG-b-[PCL-ran-PLA] block copolymer forms an injectable polyelectrolyte depot	Minocycline or sulfasalazine	Electrostatic attraction between oppositely charged drug and polymer components retards release	RA rat model; intra-articular injection	this system more closely resembles an injectable polymer depot than a conventional highly hydrated crosslinked scaffold	[90]
NanoIGUR/HA hydrogel	Acrylated HA and dithiol-terminated poly(ethylene glycol) (PEG) form a covalent network through Michael addition	Iguratimod-loaded poly(vinyl alcohol) (PVA) nanomicelles	A secondary “nanomicelle–hydrogel” diffusion barrier	CIA rats; subcutaneous injection	because the material was not administered intra-articularly, the study cannot demonstrate intra-articular retention	[69]
PRP–CS/BP hydrogel	Chitosan-based thermosensitive hydrogel	Platelet-rich plasma (PRP) and black phosphorus (BP) nanosheets	Temperature-induced gelation; BP mediates near-infrared photothermal effects, whereas PRP provides bioactive signals	RA animal model; local injection combined with near-infrared irradiation	efficacy depends on external irradiation, and the long-term safety of BP degradation and repeated irradiation requires further evaluation	[76]
IND–MTX in situ hydrogel	PEI-SS/drug nanoparticles dispersed in a 27% F127/10% F68 thermosensitive network	Indomethacin and MTX	Thermosensitive in situ gelation acts synergistically with sustained nanoparticle release to enable local dual-drug delivery	Arthritis animal model; intra-articular injection	complete in vivo concentration–time data were lacking	[91]
IND–MTX–siMMP-9 hydrogel	Thermosensitive poloxamer network containing PEI-SS composite nanoparticles	Indomethacin, MTX, and MMP-9 small interfering RNA (siRNA)	Co-delivery of small-molecule drugs and nucleic acids to simultaneously target inflammation and matrix degradation	RA animal model; intra-articular injection	the independent contribution, tissue distribution, and release synchronization of each cargo were not adequately established	[92]
IFX thermosensitive hydrogel	Thermosensitive composite network comprising F127, HA, and poly(γ-glutamic acid)	Infliximab (IFX)	Body-temperature-induced gelation retards protein diffusion	RA rabbit model; intra-articular injection	the findings should be described as sustained local delivery of a protein therapeutic and structural protection rather than cartilage regeneration	[93]
Tyr–GG hydrogel	Tyramine-modified gellan gum; HRP/H_2_O_2_-mediated enzymatic crosslinking	Betamethasone	Crosslinking density regulates swelling, degradation, and drug diffusion	Primarily material characterization and in vitro drug-release evaluation	evidence of structural efficacy in RA animals remains insufficient, and the system is more appropriately regarded as an RA-oriented delivery material	[75]
M-NO gel	Click-crosslinked network containing nitric oxide (NO)-cleavable crosslinkers and embedded self-assembled drug-loaded polymeric aggregates	Dexamethasone and NO-scavenging functional groups	Direct scavenging of excess NO; NO triggers network changes and drug release according to the degree of inflammation	RA animal model; local injection	“on-demand release” is supported by NO-response experiments, but long-term self-regulation during recurrent disease has not been validated	[94]
IFX self-healing composite scaffold	An IFX-loaded self-healing hydrogel combined with a three-dimensional (3D)-printed porous metal scaffold	IFX and adipose-derived mesenchymal stem cells	The self-healing hydrogel improves local retention of cells and biologics, whereas the metal scaffold provides structural support	RA rabbit bone-defect model; local implantation	it was used mainly to evaluate cell survival, implantation, and osteochondral repair	[12]
DNase-functionalized hydrogel	Deoxyribonuclease I (DNase I) conjugated to oxidized HA, followed by formation of a dynamic network with carboxymethyl chitosan through a Schiff base reaction	DNase I; combined with MTX in some experiments	Prolongs the local activity of DNase I and continuously degrades neutrophil extracellular traps (NETs)	CIA animal model; intra-articular injection	the evidence primarily supports NET clearance and anti-inflammatory activity and cannot independently demonstrate structural regeneration	[73]
TAP/Cx-HA hydrogel	Click-crosslinked HA hydrogel	Toll-like receptor antagonist peptide (TAP)	The covalently crosslinked network improves intra-articular peptide stability and prolongs exposure	RA animal model; intra-articular injection	structural improvement was assessed mainly using histological and matrix-related indicators, with limited evidence of functional repair	[95]
Metabolism-driven responsive hydrogel	Composite hydrogel responsive to the disease-associated metabolic environment	Psoralen and oxygen-supplying/microenvironment-modulating components	Exploits hypoxia, enzymatic activity, or metabolic state to achieve local responsiveness and drug release	RA animal model; local injection	actual pathological thresholds, interpatient variability, and reproducibility of triggering require further validation	[85]
TNF-α-binding supramolecular hydrogel	Supramolecular network based on reversible noncovalent interactions and incorporating TNF-α-binding sites	The material itself provides TNF-α-capturing functionality	Rapidly binds and locally neutralizes TNF-α rather than relying solely on drug diffusion	RA animal model; intra-articular injection	TNF-α-binding capacity, inflammatory improvement, and long-term structural modification should be evaluated as distinct outcomes	[96]
DSP–DS supramolecular hydrogel	Dexamethasone sodium phosphate and diclofenac sodium self-assemble into a gel through noncovalent interactions	Both drugs serve simultaneously as therapeutic components and gel-forming units	Drug self-assembly without an additional polymer carrier; thixotropy supports injection	AIA rats; local administration	network stability depends on drug–drug interactions, and in vivo dissociation and local pharmacokinetics remain to be defined	[5]
Anti-inflammatory–osteogenic interpenetrating network	HA–collagen interpenetrating network containing bisphosphonate-functionalized components and zinc-doped calcium phosphate	Anti-inflammatory components and bone-affinitive/mineralization-supporting components	Combines local anti-inflammatory activity, bone-surface localization, and mineralization support	RA bone-erosion model; local injection or defect filling	stable integration of newly formed bone with pre-existing erosion defects requires long-term confirmation	[6]
HP@CEL hydrogel	Supramolecular composite network formed by hydrophobically modified HA and celastrol-loaded PECT nanoparticles	Celastrol	Rapid formation of a nanodrug–hydrogel depot after injection, followed by sustained release	RA animal model; intra-articular injection	stricter component-dissection controls are needed to distinguish the relative contributions of the cargo and the material itself	[18]
Dual dynamically crosslinked TPL hydrogel	Adaptive network formed by two types of reversible dynamic bonds	Triptolide (TPL) and ROS-modulating components	The dynamic network provides injectability and self-healing; the material regulates ROS while enabling sustained drug release	RA animal model; intra-articular injection	“microenvironment-independent release” does not imply the absence of dose fluctuations and still requires confirmation by in vivo pharmacokinetic (PK) studies	[80]
Self-healing gas-regulating hydrogel	Dynamic, self-healing injectable network	Functional modules for NO scavenging and hydrogen sulfide (H_2_S) delivery	Simultaneously regulates oxidative/nitrosative stress and the gaseous-signaling microenvironment	RA animal model; intra-articular injection	gas-release dose, local concentration, and the long-term safety window remain to be quantified	[97]
Staged MTX-release hydrogel	ROS-scavenging injectable hydrogel incorporating a hierarchical drug-binding structure	MTX	Staged release comprising initial, sustained, and pathological-feedback-associated phases, together with simultaneous ROS scavenging	RA animal model; intra-articular injection	“self-regulation” should nevertheless be validated using in vivo local pharmacokinetics rather than cumulative in vitro release curves	[98]
Anti-inflammatory–antiferroptotic adhesive hydrogel	Tissue-adhesive injectable network serving as a local nanotherapeutic depot	Inflammation-modulating and antiferroptotic nanotherapeutic components	Wet-tissue adhesion improves retention while simultaneously targeting inflammation and ferroptosis-related pathways in synovial cells	RA animal model; intra-articular injection	improvement in ferroptosis-related indicators cannot substitute for structural endpoints in cartilage and bone	[99]
pH-responsive peptide hydrogel	Injectable, pH-sensitive, self-assembling peptide hydrogel	MTX and BiNS/PEI nanosheets	Responds to the acidic synovial microenvironment; MTX regulates macrophages, whereas photothermal/photodynamic effects eliminate excessive proliferation	RA animal model; intra-articular injection combined with irradiation	reliance on external irradiation raises concerns regarding damage to normal synovium and the risk of nonselective cell elimination	[100]
DAGQD@Cu@KGN–SO_3_^−^/DA-HA hydrogel	Double network comprising dopamine-modified HA and sulfonated HA	Cu single-atom nanozyme and dopamine-hybrid graphene quantum dots grafted with kartogenin (KGN)	Dopamine provides adhesion, sulfonate groups enhance hydration lubrication, the nanozyme scavenges ROS, and KGN is released continuously	CIA rats and rabbits with RA-associated osteochondral defects; intra-articular injection/defect application	“full-cycle treatment” remains limited to preclinical models	[13]
IL-4-immobilized HA hydrogel	HA-based hydrogel in which interleukin-4 (IL-4) is covalently immobilized within the network	Immobilized IL-4	Local immobilized presentation replaces rapid diffusional release and withstands repeated mechanical loading in the joint	RA animal model; intra-articular application	the local effects of an immobilized cytokine should be distinguished from the pharmacokinetic concept applicable to a releasable drug	[15]
Polymer-modified DNA hydrogel	Polymer-modified deoxyribonucleic acid (DNA) forms a gel through sequence pairing and network assembly	Functional mitochondria and nanozymes	The DNA network co-immobilizes biological organelles and catalytic components, thereby reducing local clearance	RA animal model; intra-articular injection	preservation of mitochondrial activity, batch-to-batch consistency, immunogenicity, and storage conditions are major translational barriers	[16]
ChSMA@SPD hydrogel	Methacrylated chondroitin sulfate (ChSMA) network	Spermidine (SPD)	The ECM-like network locally encapsulates SPD and modulates inflammation and chondrocyte catabolism	RA-related coculture and animal models; local application	the quality of newly formed tissue, interfacial integration, and functional recovery have not been adequately demonstrated	[52,55]
SIN liposome/PGA–F127 hydrogel	Composite hydrogel comprising poly(γ-glutamic acid) (PGA) and Pluronic F127	Sinomenine hydrochloride (SIN)-loaded liposomes	The liposomes and thermosensitive/composite gel form a secondary release barrier	RA animal model; local administration	the duration of effective intra-articular concentrations should not be inferred solely from in vitro release data	[68]
MMP-binding hydrogel	Functionalized network containing MMP-binding sites	Material-based binding sites serve as the primary functional module	Locally captures or restricts pathological MMPs, thereby reducing matrix-degradation pressure	RA animal model; intra-articular injection	MMP binding does not imply inhibition of all matrix-degrading enzyme activity	[72]
HC@PTM composite hydrogel	Dynamic Schiff base network formed by aldehyde-functionalized HA and chitosan and containing MTX-loaded polymeric micelles	MTX and ROS-scavenging/ROS-responsive micelles	The hydrogel provides injectability, self-healing, and shape adaptability; the micelles respond to ROS and pH	RA animal model; intra-articular injection	the in vivo timescales of hydrogel degradation, micelle dissociation, and MTX exposure require separate validation	[74]
Gel-MTX/Mg supramolecular hydrogel	Supramolecular network driven by structural rearrangement and noncovalent interactions	MTX and Mg^2+^	Therapeutically active components participate in network formation and enable sustained local delivery	RA animal model; intra-articular injection	the local Mg^2+^ concentration, release stability, and independent contribution of Mg^2+^ require further clarification	[7]
MOS in situ pore-forming hydrogel	Injectable, in situ pore-forming network comprising manno-oligosaccharide-modified chondroitin sulfate and HA	Manno-oligosaccharide targeting units intrinsic to the material	Pore formation promotes cell–material interactions, whereas manno-oligosaccharides act on cluster of differentiation 206 (CD206)-associated macrophages	RA animal model; intra-articular injection	its mechanism should not be reduced to a conventional binary M1/M2 polarization model	[77]
Ca^2+^-reinforced all-active hydrogel	Therapeutically active components participate in network formation, which is reinforced through in situ Ca^2+^ coordination	Network-forming units with intrinsic pharmacological activity	Reduces the proportion of inert carrier materials; Ca^2+^ enhances network stability and regulates release	RA animal model; local injection	“all-active” indicates that the components participate in treatment but does not imply a dose–response relationship or long-term safety	[101]

**Table 3:** Therapeutic Evidence Across the Synovium–Cartilage–Bone–Function Dimensions in Representative Rheumatoid Arthritis Hydrogel Studies.

Hydrogel System and Principal Intervention Target	Evidence from the Synovium and Local Immune Environment	Cartilage-Related Evidence	Bone-Related Evidence	Evidence of Pain Relief or Joint Function	Cautious Interpretation Based on Measured Endpoints	Reference
Dexamethasone-loaded HA–Tyr hydrogel	Reduced interleukin-6, prostaglandin E2 (PGE2), and multiple inflammatory cytokines in a CIA model	Cartilage thickness, type II collagen, aggrecan, and newly formed cartilage were not independently quantified	TRAP, micro-CT, and quantitative bone-erosion outcomes were not reported	No direct pain or motor-function endpoints were reported	cartilage or bone structural modification cannot be concluded solely from overall H&E findings	[4]
Infliximab-loaded F127–HA–PGA thermosensitive hydrogel	Reduced TNF-α, IL-1β, IL-6, and IL-17 levels in synovial fluid and cartilage; alleviated joint swelling and increased surface temperature	Histological findings showed reduced cartilage destruction, supporting cartilage structural protection	Bone erosion and bone-remodeling endpoints were not included	Weight-bearing index and pain-related behavior indicated pain relief	the short follow-up period and absence of bone endpoints are insufficient to demonstrate whole-joint structural modification	[93]
Indomethacin/MTX/MMP-9 siRNA in situ hydrogel	Reduced local inflammation and suppressed MMP-9-associated catabolism through siRNA	Histological and cartilage matrix-related findings indicated reduced or partially reversed cartilage destruction	Three-dimensional bone structure and bone-erosion volume were not systematically reported	Gait, weight bearing, and range of motion were not reported	“reversal of cartilage destruction” does not demonstrate the formation of mature hyaline cartilage	[92]
DNase I-functionalized injectable hydrogel	Preserved DNase I activity and promoted NET degradation; reduced the NET burden, local inflammation, and arthritis severity	Overall joint histology improved, but evaluation of cartilage-specific matrix and repair endpoints was limited	Micro-CT-based bone erosion and new bone formation were not adequately reported	No direct functional endpoints were reported	cartilage regeneration or bone repair cannot be inferred from reduced inflammation	[73]
TAP2-loaded click-crosslinked HA hydrogel	Prolonged the intra-articular retention of the TLR4-antagonistic peptide and suppressed TLR4-associated inflammatory responses and synovial pathology	Cartilage thickness, glycosaminoglycan preservation, and histological improvement were reported	Bone-related histological changes were reported, but adequate long-term evidence of three-dimensional bone reconstruction was lacking	No direct functional endpoints were reported	functional evaluation and long-term integration remain lacking	[95]
HP@CEL HA–nanodrug supramolecular hydrogel	Regulated macrophage–FLS crosstalk through sustained celastrol release and suppressed proinflammatory macrophage states, FLS activation, and inflammation	Cartilage histology and ECM-related indicators improved	Improvements in joint bone structure were reported, but independent quantitative evidence of bone repair was limited	Gait, weight bearing, and joint range of motion were not reported	sustained and functional regeneration of cartilage or bone has not been demonstrated	[18]
SPT@TPL dual dynamically crosslinked hydrogel	Scavenged or regulated excessive ROS, reduced inflammation, and promoted the transition of macrophages toward repair-associated states	Improvements in cartilage-surface morphology and matrix staining indicated cartilage protection or early repair	Bone structure was not a principal validation endpoint	No direct functional endpoints were reported	histological improvement alone should not be described as functional cartilage regeneration	[80]
Anti-inflammatory–osteogenic interpenetrating-network hydrogel	Regulated local inflammation and the macrophage-associated osteoimmune microenvironment	Cartilage was not the primary repair target, and cartilage-specific outcomes were relatively limited	Osteoclast activity, osteogenic differentiation, and micro-CT bone structure were evaluated	The direct mechanical function of newly formed bone and recovery of overall joint movement were not reported	an experimentally created erosion defect is not fully equivalent to naturally progressive bone erosion in RA	[6]
DNRS dual-gas-regulating self-healing hydrogel	Scavenged excessive NO, released H_2_S, regulated macrophages and the inflammatory microenvironment, and reduced synovial inflammation	Cartilage was not the principal repair endpoint, and structural evidence was limited	TRAP staining and micro-CT indicated inhibition of osteoclast activity and improvement in bone structure	Direct assessments of joint mechanics, gait, and range of motion were not reported	the quality of newly formed bone, integration with host tissue, and stability after inflammatory recurrence require further validation	[97]
Adhesive and lubricating DAGQD@Cu@KGN double-network hydrogel	The Cu single-atom nanozyme scavenged ROS and reduced local inflammatory responses	Reduced friction and cartilage wear in an early-stage model	Bone was not a principal assessment target, and a complete evidence chain for bone-erosion repair was not established	Material tribological properties were evaluated, but adequate evidence from animal gait, weight-bearing, and joint-range-of-motion assessments was lacking	reduced friction in vitro cannot substitute for restoration of whole-joint function	[13]
MMP-9-binding CuS-T/ChSMA hydrogel	Locally bound MMP-9, inhibited RA-FLS invasion and proinflammatory macrophage states, and alleviated synovial inflammation	Increased expression of type II collagen- and aggrecan-related markers	Bone erosion and new bone formation were not adequately evaluated	No direct functional endpoints were reported	mature cartilage regeneration cannot be concluded solely from collagen expression and improved staining	[72]
Polymer-modified DNA hydrogel co-delivering functional mitochondria and Prussian blue nanozymes	Reduced oxidative stress and inflammation through extracellular ROS scavenging and intracellular mitochondrial renewal	Improvements in cartilage histology, chondrocyte status, and matrix repair-associated indicators were reported	Improvements in bone and osteochondral structures were reported, but long-term bone reconstruction and mechanical quality remain unclear	Standardized recovery of gait or weight bearing was not reported	preservation of mitochondrial potency, immune safety, and batch-to-batch consistency remain critical limitations	[16]
Spermidine-loaded ChSMA hydrogel	Reduced local inflammation and modulated inflammation-associated cells and the osteoimmune microenvironment	Reduced chondrocyte apoptosis and MMP expression and improved type II collagen, aggrecan, and cartilage histology	Suppressed osteoclast-related signaling, improved bone-erosion or bone-microstructural indicators, and regulated the osteoblast–osteoclast balance	No direct functional endpoints were reported	the mechanical properties of newly formed tissue, long-term integration, and joint function remain unproven	[52]
Gel-MTX/Mg supramolecular drug-loaded hydrogel	Reduced inflammatory mediators and clinical arthritis scores and modulated the local immune state	Preservation of the cartilage surface and matrix was reported	Suppressed osteoclast differentiation and improved indicators of bone erosion and bone microstructure	Standardized functional endpoints were not reported	evidence remains insufficient to conclude that erosion defects underwent stable bone regeneration	[7]
IL-4-covalently immobilized immunosuppressive hydrogel	Immobilized IL-4 exerted sustained effects on local macrophages and reduced proinflammatory states and synovial inflammation	Reduced cartilage degradation or joint-tissue damage was reported, primarily indicating structural protection	Bone erosion and new bone formation were not adequately validated	No direct functional endpoints were reported	changes in macrophage phenotype should not be equated with complete restoration of synovial homeostasis	[15]
Infliximab-loaded self-healing hydrogel–porous metal composite scaffold delivering adipose-derived stem cells (ADSCs)	Reduced local inflammatory pressure and provided a more favorable microenvironment for transplanted cells	Improvements in tissue repair and cell engraftment in the osteochondral region were reported	The scaffold supported the bone defect, and osteogenesis, implantation, and bone-tissue repair were reported	Recovery of overall RA joint mobility and load-bearing function was not adequately demonstrated	its conclusions should not be extrapolated to diffuse structural damage in RA	[12]

## Data Availability

No new data were created or analyzed in this study.

## Abbreviations

The following abbreviations are used in this manuscript:

ACAN	Aggrecan
ADAMTS	A disintegrin and metalloproteinase with thrombospondin motifs
ADAMTS-5	A disintegrin and metalloproteinase with thrombospondin motifs 5
ADSCs	Adipose-derived stem cells
AIA	Adjuvant-induced arthritis
ALP	Alkaline phosphatase
ASK1	Apoptosis signal-regulating kinase 1
BMP-2	Bone morphogenetic protein 2
BMP-7	Bone morphogenetic protein 7
BMP9	Bone morphogenetic protein 9
BP	Black phosphorus
Bregs	Regulatory B cells
BV/TV	Bone volume/tissue volume
CCL18	C-C motif chemokine ligand 18
CD80	Cluster of differentiation 80
CD86	Cluster of differentiation 86
CD147	Basigin/cluster of differentiation 147
CD206	Cluster of differentiation 206
CFU/g	Colony-forming units per gram
CIA	Collagen-induced arthritis
COL I	Type I collagen
COL II	Type II collagen
COL2A	Type II collagen alpha chain marker
CQAs	Critical quality attributes
CSF1	Colony-stimulating factor 1
CTSK	Cathepsin K
DCs	Dendritic cells
DMARD/DMARDs	Disease-modifying antirheumatic drug(s)
DNA	Deoxyribonucleic acid
DNase I	Deoxyribonuclease I
ECM	Extracellular matrix
EVs	Extracellular vesicles
FAPI	Fibroblast activation protein inhibitor
FK506	Tacrolimus
FLS	Fibroblast-like synoviocytes
GelMA	Gelatin methacryloyl
HA	Hyaluronic acid
HAPLN1	Hyaluronan and proteoglycan link protein 1
H&E	Hematoxylin and eosin
HRP	Horseradish peroxidase
H_2_O_2_	Hydrogen peroxide
H_2_S	Hydrogen sulfide
IDO	Indoleamine 2,3-dioxygenase
IFX	Infliximab
IGF-1	Insulin-like growth factor 1
IL-1β	Interleukin-1β
IL-4	Interleukin-4
IL-6	Interleukin-6
IL-17	Interleukin-17
IL-32	Interleukin-32
IL-6/JAK2/STAT3	Interleukin-6/Janus kinase 2/signal transducer and activator of transcription 3
IND	Indomethacin
JAK2	Janus kinase 2
JNK	c-Jun N-terminal kinase
KGN	Kartogenin
MAPK	Mitogen-activated protein kinase
METTL3	Methyltransferase-like 3
m6A	N6-methyladenosine
miRNA/miRNAs	MicroRNA(s)
miR-124-3p	MicroRNA-124-3p
miR-140	MicroRNA-140
miR-195a	MicroRNA-195a
miR-221/222	MicroRNA-221/222
circRNA_28313	Circular RNA 28313
MMP/MMPs	Matrix metalloproteinase(s)
MMP-1	Matrix metalloproteinase-1
MMP-2	Matrix metalloproteinase-2
MMP-3/MMP3	Matrix metalloproteinase-3
MMP-9	Matrix metalloproteinase-9
MMP-13	Matrix metalloproteinase-13
MRI	Magnetic resonance imaging
MSCs	Mesenchymal stem cells
MTX	Methotrexate
mRNA	Messenger RNA
NET/NETs	Neutrophil extracellular trap(s)
NETosis	Neutrophil extracellular trap formation
NF-κB	Nuclear factor-κB
NFATc1	Nuclear factor of activated T cells 1
NLRP3	NOD-like receptor family pyrin domain-containing 3
NO	Nitric oxide
NSAIDs	Nonsteroidal anti-inflammatory drugs
OA	Osteoarthritis
OARSI	Osteoarthritis Research Society International
OCN	Osteocalcin
OIA	Osteoarthritis-induced arthritis/osteochondral-injury-associated arthritis model, as used in the manuscript context
OPG	Osteoprotegerin
OVA/CFA	Ovalbumin/complete Freund’s adjuvant
PAD4	Peptidyl arginine deiminase 4
PD-L1	Programmed death-ligand 1
PGE_2_	Prostaglandin E_2_
PK	Pharmacokinetic(s)
PLXNC1	Plexin C1
PRG4	Proteoglycan 4
PRP	Platelet-rich plasma
PVA	Poly(vinyl alcohol)
RA	Rheumatoid arthritis
RA-FLS	Rheumatoid arthritis-derived fibroblast-like synoviocytes
RANK	Receptor activator of nuclear factor-κB
RANKL	Receptor activator of nuclear factor-κB ligand
RANKL/OPG	RANKL/osteoprotegerin ratio or balance
RANKL/TNF	RANKL/tumor necrosis factor-related strategy
RGD	Arginine–glycine–aspartic acid
RNA	Ribonucleic acid
ROS	Reactive oxygen species
RUNX2	Runt-related transcription factor 2
SAL	Sterility assurance level
SIN	Sinomenine hydrochloride
SIS	Small intestinal submucosa
siRNA	Small interfering RNA
SOX9	SRY-box transcription factor 9
SPARCL1	Secreted protein acidic and rich in cysteine-like 1
SPD	Spermidine
STAT3	Signal transducer and activator of transcription 3
TAP	Toll-like receptor antagonist peptide
Tb.N	Trabecular number
Tb.Sp	Trabecular separation
Tb.Th	Trabecular thickness
TGF-β	Transforming growth factor-β
TGF-β3	Transforming growth factor-β3
Tfh	T follicular helper cell
Th17	T helper 17 cell
TLR4	Toll-like receptor 4
TLRs	Toll-like receptors
TNF	Tumor necrosis factor
TNF-α	Tumor necrosis factor-α
TNFR1	Tumor necrosis factor receptor 1
TNFR2	Tumor necrosis factor receptor 2
TPL	Triptolide
TRAF	Tumor necrosis factor receptor-associated factor
TRAP	Tartrate-resistant acid phosphatase
Tregs	Regulatory T cells
Tr1 cells	Type 1 regulatory T cells
VEGF	Vascular endothelial growth factor
W9	W9 peptide
WYRGRL	Cartilage-binding peptide sequence WYRGRL
c-Fos	Fos proto-oncogene
c-Src	Cellular Src tyrosine kinase
micro-CT	Micro-computed tomography
Material/system abbreviation	Full term/explanation
BiNS/PEI	BiNS/polyethyleneimine nanosheets
CeNZs	Cerium-based nanozymes/ceria nanozymes
ChSMA	Methacrylated chondroitin sulfate
CS/BP	Chitosan/black phosphorus
CuS-T	MMP-9-binding CuS-T component
Cx-HA	Click-crosslinked hyaluronic acid
Cx-SIS	Click-crosslinked small intestinal submucosa
DA-HA	Dopamine-modified hyaluronic acid
DAGQD	Dopamine-hybrid graphene quantum dots
DAGQD@Cu@KGN–SO_3_^−^/DA-HA	DAGQD-, Cu-, KGN-, sulfonate-, and DA-HA-based multifunctional hydrogel
DNRS	Dual-gas-regulating self-healing hydrogel
DS	Diclofenac sodium
DSP	Dexamethasone sodium phosphate
F127	Pluronic F127/poloxamer 407
F68	Pluronic F68/poloxamer 188
Gel-MTX/Mg	Methotrexate- and magnesium-containing supramolecular hydrogel
GG	Gellan gum
HA–Tyr	Tyramine-modified hyaluronic acid
HC@PTM	Composite hydrogel containing MTX-loaded polymeric micelles
HP@CEL	Hydrophobically modified HA/celastrol-loaded nanoparticle hydrogel
HPAP	HPAP hydrogel system
IND–MTX	Indomethacin–methotrexate
IND–MTX–siMMP-9	Indomethacin–methotrexate–MMP-9 siRNA system
KGN@P407	Kartogenin-loaded Pluronic P407 system
M-NO	Nitric oxide-responsive or NO-scavenging gel system
MOS	Manno-oligosaccharide-modified system
NanoIGUR	Iguratimod-loaded nanomicelles
PCL	Polycaprolactone
PECT	PECT nanoparticles
PEG	Polyethylene glycol
PEGDMA	Poly(ethylene glycol) dimethacrylate
PEI	Polyethyleneimine
PEI-SS	Disulfide-containing polyethyleneimine component
PGA	Poly(γ-glutamic acid)
PLA	Polylactic acid/polylactide
PRP–CS/BP	Platelet-rich plasma–chitosan/black phosphorus hydrogel
SPT@TPL	Triptolide-loaded dynamically crosslinked hydrogel system
TAP/Cx-HA	Toll-like receptor antagonist peptide-loaded click-crosslinked HA hydrogel
TAP2 + Cx-HA	TAP2-loaded click-crosslinked HA system
Tyr–GG	Tyramine-modified gellan gum
